# An improved adaptive neuro fuzzy inference system model using conjoined metaheuristic algorithms for electrical conductivity prediction

**DOI:** 10.1038/s41598-022-08875-w

**Published:** 2022-03-23

**Authors:** Iman Ahmadianfar, Seyedehelham Shirvani-Hosseini, Jianxun He, Arvin Samadi-Koucheksaraee, Zaher Mundher Yaseen

**Affiliations:** 1 Department of Civil Engineering, Behbahan Khatam Alanbia University of Technology, Behbahan, Iran; 2 Department of Chemical Engineering, Science and Research Branch, Islamic Azad University, Tehran, Iran; 3 Department of Civil Engineering, University of Calgary, Calgary, AB Canada; 4 Adjunct Research Fellow, USQ’s Advanced Data Analytics Research Group, School of Mathematics Physics and Computing, University of Southern Queensland, QLD 4350 Toowoomba, Australia; 5 New Era and Development in Civil Engineering Research Group, Scientific Research Center, Al-Ayen University, Thi-Qar, 64001 Iraq

**Keywords:** Environmental chemistry, Environmental monitoring, Environmental sciences, Engineering

## Abstract

Precise prediction of water quality parameters plays a significant role in making an early alert of water pollution and making better decisions for the management of water resources. As one of the influential indicative parameters, electrical conductivity (EC) has a crucial role in calculating the proportion of mineralization. In this study, the integration of an adaptive hybrid of differential evolution and particle swarm optimization (A-DEPSO) with adaptive neuro fuzzy inference system (ANFIS) model is adopted for EC prediction. The A-DEPSO method uses unique mutation and crossover processes to correspondingly boost global and local search mechanisms. It also uses a refreshing operator to prevent the solution from being caught inside the local optimal solutions. This study uses A-DEPSO optimizer for ANFIS training phase to eliminate defects and predict accurately the EC water quality parameter every month at the Maroon River in the southwest of Iran. Accordingly, the recorded dataset originated from the Tange-Takab station from 1980 to 2016 was operated to develop the ANFIS-A-DEPSO model. Besides, the wavelet analysis was jointed to the proposed algorithm in which the original time series of EC was disintegrated into the sub-time series through two mother wavelets to boost the prediction certainty. In the following, the comparison between statistical metrics of the standalone ANFIS, least-square support vector machine (LSSVM), multivariate adaptive regression spline (MARS), generalized regression neural network (GRNN), wavelet-LSSVM (WLSSVM), wavelet-MARS (W-MARS), wavelet-ANFIS (W-ANFIS) and wavelet-GRNN (W-GRNN) models was implemented. As a result, it was apparent that not only was the W-ANFIS-A-DEPSO model able to rise remarkably the EC prediction certainty, but W-ANFIS-A-DEPSO (R = 0.988, RMSE = 53.841, and PI = 0.485) also had the edge over other models with Dmey mother in terms of EC prediction. Moreover, the W-ANFIS-A-DEPSO can improve the RMSE compared to the standalone ANFIS-DEPSO model, accounting for 80%. Hence, this model can create a closer approximation of EC value through W-ANFIS-A-DEPSO model, which is likely to act as a promising procedure to simulate the prediction of EC data.

## Introduction

### Research background

Nowadays, water resources face plenty of serious threats, all of which are caused mainly by the increasing phenomenon of climate change, urbanization and inadequate water infrastructure^1^. Undoubtedly, rivers are vital inland water resources to supply a wide range of purposes such as agricultural demands, industrial and recreational goals, and domestic consumption^2,3^. Accordingly, the essential role of existing water resources, in particular rivers, emphasizes the necessity of efficient water resource management^4^. Admittedly, the primary key of effective water resource management is to monitor the water quality (WQ) on a regular basis^5–8^.

Nevertheless, frequent and accurate testing and sampling of existing water bodies are time-consuming and exorbitant, resulting in the tedious study and limited calibration and validation of WQ evaluation^9,10^. Developing appropriate data-intelligence models for WQ monitoring is part and parcel of better surface water management^11,12^. Hence, the main benefit of modeling the water quality-related variables of surface water is to reach an efficient and reliable water resource management, which causes costs reduction. It is mainly because these models, as indirect procedures, have high reliability, which would detect the values of water quality-related variables in the future^13^. Ensuring better WQ management demands the development of suitable models for WQ monitoring^14–17^. As a result, reaching a better understanding of surface water-related parameters is one of the significant elements to have reliable water resources management^18^. It can result in a more efficient emerging tool of water treatment cost reduction and better WQ sustainability.

Among several WQ variables, EC is an essential indicator for salinity that is highly significant for better irrigation and water usages purposes^19^. This is clearly explaining the importance of this variable in the surface water quality health as it is prevailed by the total dissolved solid (TDS) while being related to dissolved ionic solutes including sodium (Na^+^), chloride (Cl^−^), magnesium (Mg^2+^), sulfate (SO4^2−^), and calcium (Ca^2+^) in water^5,19^. Not only does the ionic composition affect the growth of plants, but it also decreases the quality of drinking water remarkably. Besides, EC plays an essential role in salinity hazard measurement for irrigation and drinking water^5,19,20^.

### Problem statement

Water quality variables models are critically important tools for conducting aquatic systems research, bringing along an appropriate evaluation and prediction of surface WQ for effective water resource management^7,21–24^. Indeed, this procedure causes efficient measures to ensure that pollution proportions remain within permissible limits^25^. One of the essential tasks to reach optimal resource management is to predict the WQ parameters accurately. Although the traditional process-based modeling methods lead to accurate WQ parameters prediction, these models have some restrictions^3,26–29^. They are limited to single particular catchment, certain type of data stochasticity and data redundancy. As an illustration, they work based on data set requiring a great deal of processing time and unknown input data. Furthermore, since WQ is affected by distinct parameters, conventional strategies for data processing do not have enough efficiency in solving this problem, and these parameters illustrate a sophisticated non-linear relationship with parameters of WQ prediction^30^.

### Literature review predictive models

In recent years, various modelling procedures have taken place to enhance the prediction accuracy of diverse WQ models^3^. For example, diverse mathematical models based on statistical perspectives were established i.e., linear regression model^31^, moving average (MA) and autoregressive integrated moving average (ARIMA)^32,33^. The forgoing models have been developed and employed to predict water quality; however, they give rise to some obstacles. Some drawbacks of statistical models are that they use linear and normally distributed relationships between the prediction and response^26^. Moreover, these models are unlikely to supply accurate predictions owing to the shortage of authentic tools to gather observation data for the timeframe, the complexity of influential criteria in prediction, and the shortcoming to receive non-stationarity and nonlinearity of the WQ parameters^34^. Nowadays, using artificial intelligence methods can significantly assist in increasing the model’s accuracy and reliability^35–38^. Using machine learning (ML) models are experiencing an increasing trend in solving environmental problems, which stems from their striking ability to solve sophisticated non-linear problems^11,14,28,39–42^, and their non-reliance on pre-knowledge of the physical processes, although these ML models need large data volumes to work properly. Since the ML technique is an influential procedure to model sophisticated non-linear systems, it promotes the development of parallel computing and computational capabilities significantly, which causes researchers to operate ML methods^5^.

Recently, to address free ammonia (AMM), total Kjeldahl nitrogen (TKN), water temperature (WT), total coliform (TC), fecal coliform (FC), and pH, the least square support vector machine (LSSVM), multivariate adaptive regression splines (MARS), and M5 model tree (M5Tree) were conducted^43^. The outcomes of MARS and LSSVM models proved the higher ability in comparison with other methods. Adaptive neuro fuzzy inference system (ANFIS) was developed to forecast the WQ parameters in Manzala Lake by Ref.^44^, which led to an accurate WQ parameters prediction. Eventually, it detected the total nitrogen and phosphorus contents in the region. The mixture of artificial neural network (ANN) model and multi-objective genetic algorithm (MOGA) was employed by Ref.^45^ to enhance WQ prediction performance, which brought along better capability.

Recently, the hybrid wavelet-artificial neural network (WANN) procedures forecasted the EC of river water^20^ to assess the WQ parameters based on limited time series data,, which revealed the WANN model led to modified modeling. Barzegar et al.^39^ applied ANN, ANFIS, wavelet-ANN, and wavelet-ANFIS to evaluate water salinity levels according to Ca^2+^, Mg^2+^, Na^+^, SO4^2−^, and Cl^−^ in rivers on a monthly basis. Ravansalar et al.^8^ enhanced a new hybrid wavelet-linear genetic programming (WLGP) model to forecast sodium (Na^+^) concentration every month, bringing along the remarkable ability of the WLGP model in terms of prediction of the Na^+^ peak values. Furthermore, Barzegar et al.^6^ modelled multi-step-ahead EC via a hybrid wavelet-extreme learning machine (WA-ELM) model, being compared with an adaptive neuro-fuzzy inference system (ANFIS). Li et al.^15^ combined recurrent neural network (RNN) with modified Dempster/Shafer (D–S) evidence theory to create a hybrid RNNs-DS model. Jafari et al.^14^ used a hybrid wavelet-genetic programming (WGP) model for improved water biochemical oxygen demand (BOD) prediction. Indeed, this model was assessed against 5 ML, having W-ANN, ANN, GP, DT, and BN, outperforming the comparative models. Najah Ahmed et al.^26^ presented a Neuro-Fuzzy Inference System (WDT-ANFIS) based on an augmented wavelet de-noising manner, dependent on historical data of the WQ parameter. They used three evaluation techniques to address diverse influences on the model. Finally, it revealed the proposed model could forecast all WQ parameters.

In hybrid models, Deng et al.^34^ investigated a multi-factor WQ time series prediction model based on Heuristic Gaussian cloud transformation, which led to an enhanced model for forecasting accurately. Zhou et al.^24^ improved grey relational analysis (IGRA) algorithm and long-short term memory (LSTM) neural network-based model for WQ prediction, resulting in a noteworthy performance of the proposed model in WQ prediction compared to the benchmarked models. Haji Seyed Asadollah et al.^3^ introduced a new ensemble machine learning model, extra tree regression (ETR), to forecast the water quality index (WQI) amounts at the Lam Tsuen River in Hong Kong on a monthly basis. Comparing this model with classic standalone models, support vector regression (SVR) and decision tree regression (DTR), they found the ETR model led to more authentic WQI predictions for both training and testing phases. Dehghani et al.^46^ introduced a hybrid of grey wolf optimizer (GWO) with ANFIS model to forecast multi-ahead influent flow rate. Their results indicated that the proposed model could reliably estimate the influent flow rate from 5-min up to 10 days. For the prediction of the dissolved oxygen (DO) at two stations in Yangtze River, China, an improved least square support vector machine (LSSVM) coupled with the sparrow search algorithm (SSA) was introduced by Ref.^47^. In addition, the variational mode decomposition (VMD) was applied to denoise the input dataset. Their results indicated that the proposed model has better efficiency than standalone LSSVM, VMD-LSSVM, and SSA-LSSVM to predict the DO. The literature reviews, highly emphasized the implication of hybrid machine learning models for diverse environmental engineering problems^48–50^. It has been approved as those newly developed versions are the trustworthy computer aid models for solving highly stochastic and non-linear historical big data^51,52^.

### Main objectives and contributions

Over the past decade, researchers, particularly hydrologists, have witnessed a remarkable increment trend around the world to discover influential computational models for surface WQ simulation^53,54^. It has been concomitant with noteworthy advancements in modeling. Needless to say, understanding the surface WQ perfectly, as a natural problem, is a challenging issue. In turn, the key goal of devising a novel hybridized version of ML models is to assess this ordeal more effectively. The necessity of the internal model parameters tuning, data clustering and cleaning, data preprocessing and several others gave rise to some limits in Standalone ML model. Hence, the current study is motivated to develop an efficient hybrid model coupled with wavelet theorem, called wavelet adaptive neural fuzzy inference system couple with an adaptive hybrid of differential evolution and particle swarm optimization (W-ANFIS-A-DEPSO). In fact, the main contribution of this study is to hybridize the ANFIS model with an efficient optimization method called A-DEPSO, which is a novel hybrid model u. The A-DEPSO algorithm uses a powerful local and global search mechanism to avoid local solutions and moves toward the global solution. In addition, it uses an adaptive control parameter to assist the algorithm in balancing the exploration and exploitation. The model is designed to predict EC index on a monthly basis at Maroon River, Iran. Accordingly, a set of data including monthly discharge (Q) and EC measured over three decades, from 1980 to 2016, at Tange-Takab station is used for modeling.

A set of preprocessing analyzes, being essential in the proposed model training process, is intended to select the appropriate input parameters to the predictive models. In order to address the most excellent selective combinations separately for EC, the best subset regression approach is planned. By considering a wide range of statistical metrics, graphical implements, and error analysis in selective combinations, the predictive abilities for the standalone and wavelet-based ML models are evaluated.

## Methodology

### Adaptive neuro fuzzy inference system (ANFIS)

**Figure 1:**
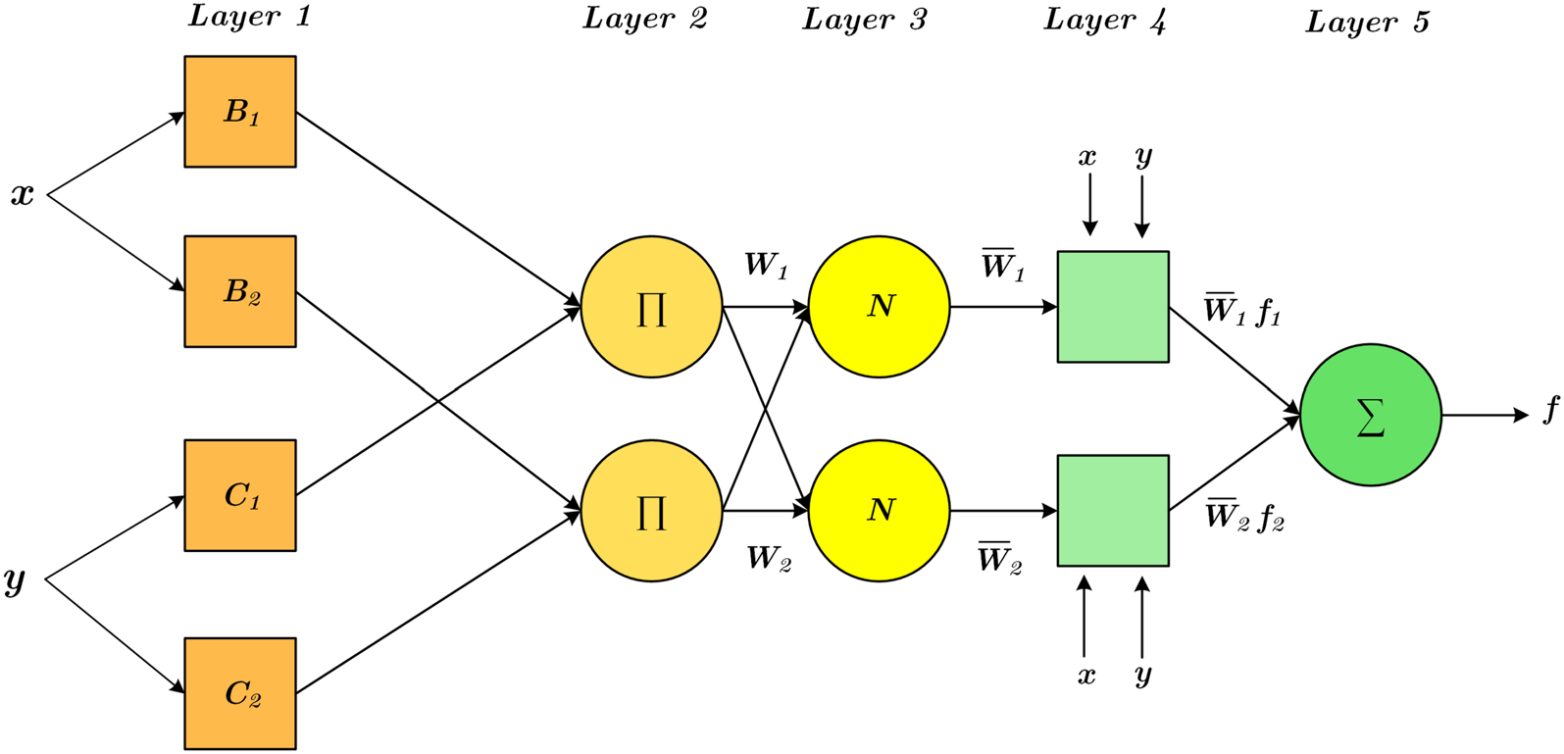
Schematic of ANFIS model.

ANFIS is a hybrid method merging the ANN and fuzzy logic, which is first initiated by Ref.^55^. ANFIS uses the IF–THEN fuzzy rules (FRs) for describing the knowledge between the input and target dataset of a modeling problem^56,57^. The main structure of the ANFIS model is displayed in Fig. 1. In this model, the Takagi–Sugeno inference procedure plays an integral role in generating the if–then rules, the range from input to output.

1 $$ {\text{Rule1}}:{\text{ if}}\,x\,{\text{is}}\,D_{1} \,{\text{and}}\,{\text{y}}\,{\text{is}}\,C_{1} \,,{\text{ then}}\,g_{1} = \alpha_{1} x + \beta_{1} y + \gamma_{1} , $$

2 $$ {\text{Rule2}}:{\text{ if}}\,x\,{\text{is}}\,D_{2} \,{\text{and}}\,{\text{y}}\,{\text{is}}\,C_{2} \,,{\text{ then}}\,g_{2} = \alpha_{2} x + \beta_{2} y + \gamma_{2} $$

in which ${\alpha }_{1}$, ${\beta }_{1}$, ${\gamma }_{1}$, and ${\alpha }_{2}$, ${\beta }_{2}$, ${\gamma }_{2}$ denote the consequence parameters, and ${D}_{1}$, ${D}_{2}$, ${C}_{2}$, and ${C}_{2}$ are considered membership functions (MFs). The ANFIS includes five layers, with it consisting of a wide range of inputs and just one output. This structure is elaborated as follows:

During the first layer, each node is controlled by a specific function parameter, which is then used to generate an amount of membership degree ($\psi$) by the bell MF.

3 $${Q}_{1k}={\psi }_{Dk}\left(x\right), k=\mathrm{1,2},$$

4 $$\begin{array}{cc}& {Q}_{1k}={\psi }_{{C}_{k-2}}\left(y\right), k=\mathrm{3,4}\\ & \psi (x)=\frac{1}{1+{\left[{\left(\frac{x-{e}_{k}}{{a}_{k}}\right)}^{2}\right]}^{{m}_{k}}},k=\mathrm{1,2}\end{array}$$

in which ${a}_{k}$, ${e}_{k}$, and ${m}_{k}$ are defined as membership values. At the second layer, each node output is regarded an input signal, indicating the firing strength of each rule.

5 $${Q}_{2k}={\psi }_{{D}_{k}}\left(x\right)\times {\psi }_{{D}_{-2}}\left(y\right).$$

Concerning layer 3, the strength ratio for *k*th rule to all rules' sum strength is estimated by the output of layer 3.

6 $$ Q_{3k} = \overline{w}_{k} = \frac{{\omega_{k} }}{{\mathop \sum \nolimits_{k = 1}^{2} \omega_{k} }} $$

In addition, the adaptive nodes can be estimated in layer 4.

7 $$ Q_{4k} = \overline{w}_{k} g_{k} = \overline{w}_{k} \left( {\alpha_{k} x + \beta_{k} y + \gamma_{k} } \right). $$

Eventually, layer 5 computes the network output.

8 $${Q}_{5\mathrm{k}}=\sum_{k} {w}_{k}{g}_{k}.$$

### Least square support vector machine (LSSVM)

Suykens and Vandewalle introduced a new method of the support vector machine (SVM) called LSSVM, which uses linear equations to raise the convergence speed^58–60^. However, the SVM works with a quadratic programming technique for training^61^. To put it simply, in terms of simple structure and high convergence speed, the LSSVM has the edge over SVM, which results in more popular methods in regression and classification fields^61,62^. In the following, the model was formulated in which the training dataset is defined through (${x}_{n}$, ${y}_{n}$), *n* = 1, 2, …, *N*, as ${x}_{n}$ and ${y}_{n}$ are considered the input and output dataset.

9 $$ Minimize_{{\psi ,{\text{b}},\zeta }} F(\psi {{,\zeta ) = }}\frac{1}{2}\psi^{T} \psi + \frac{1}{2}\lambda \mathop \sum \limits_{i = 1}^{N} \lambda_{i}^{2} , $$

$$ Subject\,to: $$

10 $$ y_{n} = \psi^{T} \theta \left( {x_{n} } \right) + b + {\upzeta }_{n} {, }n = 1{,} 2{,} \ldots {,} N, $$

where $\lambda$ is considered a penalty parameter, ${\upzeta }$ is defined as a regression error, $\theta \left( {x_{m} } \right)$ is a non-linear function,$\psi^{T}$ is the transposed output layer vector, $b$ is a parameter for calculation. In addition, the Eqs. (9) and (10) may be expressed by the Lagrange procedure. So,

11 $$ L(\psi {\text{, b}}, \zeta , \beta {) = }Z\left( {\psi {{,\zeta )}} - \mathop \sum \limits_{n = 1}^{N} \beta_{n} (\psi^{T} \theta \left( {x_{n} } \right) + b + {\upzeta }_{n} - y_{n} } \right). $$

In which ${\beta }_{n}$ is defined as a lagrange multiplier. Through Karush–Kuhn–Tucker (KKT) conditions, some solutions are gained, and in the following, they are formulated:

12 $$ \frac{\partial L}{{\partial \psi }} = 0 \to \psi = \mathop \sum \limits_{n = 1}^{N} \beta_{n} \theta \left( {x_{m} } \right), $$

13 $$ \frac{\partial L}{{\partial b}} = 0 \to \mathop \sum \limits_{n = 1}^{N} \beta_{n} = 0, $$

14 $$ \frac{\partial L}{{\partial {\upzeta }_{n} }} = 0 \to \beta_{n} = \lambda {\upzeta }_{n} , $$

15 $$ \frac{\partial L}{{\partial \beta_{n} }} = 0 \to \psi^{T} \theta \left( {x_{n} } \right) + b + {\upzeta }_{n} - y_{n} = 0. $$

The linear equations mentioned below may be gained by solving $\psi$ and ${\upzeta }_{n}$ parameters.

16 $$\left[\begin{array}{cc}0& {g}_{n}^{T}\\ {g}_{n}& Krl+{\lambda }^{-1}e\end{array}\right]\left[\begin{array}{c}b\\ \beta \end{array}\right]=\left[\begin{array}{c}0\\ y\end{array}\right]$$

in which ${g}_{n}={[1,\dots , 1]}^{T}$, $\beta ={{[\beta }_{1}, \dots , {\beta }_{n}]}^{T}$, $y={{[y}_{1}, \dots , {y}_{n}]}^{T}$ And $g$ is regarded as the unit matrix. $Krl$ being the kernel functions are formulated as,

17 $$K\left({x}_{m},{x}_{i}\right)=\theta \left({x}_{m}\right)\theta \left({x}_{i}\right).$$

Radial basis functions (RBF) are operated as the kernel function as:

18 $$Krl\left({x}_{n}\text{,} {x}_{j}\right)=\mathrm{exp}\left(\frac{-\left|\left|{x}_{n}, {x}_{j}\right|\right|}{{\gamma }^{2}}\right)$$

in which $\delta$ being a fixed parameter.

### Generalization regression neural network (GRNN)

In 1991, a probabilistic-based neural network based on radial basis function (RBF), known as Generalized regression neural network (GRNN), was introduced by Specht^63^. Nowadays, this model is usually used for classification and regression while dealing with non-linear fitting systems in large-scale samples, and the model operates according to the nonparametric kernel regression network. Overall, GRNN may lead to a fewer local minimum by a learning algorithm, whereas it has fewer adaptation parameters by comparison with the backpropagation and RBF artificial neural network^64^. In other words, this model accounts for four layers, input layer, radial layer, regression layer, and an output layer, with the structure consisting of a radial neurons layer and a regression layer which are located in the input and output layers^65^. To add to it, pattern (radial neurons) layer in which there is the input data in training step, the neurons number is identical with the data sample points. Besides, the summation layer has provided by a different neuron rather than the output layer being considered to estimate the density function. However, other neurons are supplied with the purpose of output estimation. To sum up, the GRNN model spend less time operation in comparison with other ANNs, since this method has directly selection operation between predictors and target^65^. This model uses a control parameter called spread parameter, exhibits the spread of RBF and regulates the function to obtain the most relevant fitness.

### Multivariate adaptive regression spline (MARS)

The MARS method is an innovative side of stepwise linear regression (SLR) and is used to solve modeling problems having high input parameters, and it was presented by Friedman^66^. Regarding the MARS operation, this method works differently, as it brings about diverse slopes (i.e. linear bias functions (LBFs)) for different domains of variable range, whilst the SLR utilizes one slop for input variable^67^. Therefore, it can be concluded that the MARS can be concomitant with more data than the SLR to elaborate on how an essential variable impacts the dependent variable. Consequently, the MARS is made based on the LBFs structure and stems from the SLR classification^68^. This means it is unlikely to need previous knowledge to determine LBF numbers and parameters. In turn, it can be expressed that the MARS method has the edge over SLR thanks to the mentioned merit. Also, it is noteworthy that through a set of elementary LBFs the connection between input and output data appears, and in the following a LBF is formulated,

19 $${D}_{n}\left(x\right)=\mathrm{max}\left(0, B-x\right) or {D}_{n}\left(x\right)=\mathrm{max}\left(0, x-B\right)$$

in which $B$ is a beginning variable in order to divide the $x$ range into sub-ranges, ${D}_{n}$ is a basic function, $x$ defines the input dataset. The fundamental MARS formulation is expressed as,

20 $$ G\left( x \right) = \alpha_{0} + \mathop \sum \limits_{n = 1}^{N} \alpha_{n} D_{n} \left( x \right), $$

where $G\left(x\right)$ demonstrates the output, *N* is the total number of weighting factors, and ${\alpha }_{0}, {\alpha }_{1}, \dots , {\alpha }_{N}$ are weighting factors in the MARS method.

There are two fundamental steps in the MARS application process: forward and backward stages. The forward is a step in which it is tried to reduce the probable errors in the training phase by increasing LBFs in the model. Eventually, this step is completed by the provided total number of LBFs. On the other hand, the second stage is to decrease the overfitting trend, with it eradicting the extra LFBs. Estimating sub-models are necessary, being done using the generalized cross-validation (GCV) index^69,70^ so,

21 $$GCV=\frac{MSE}{(1-\frac{m+0.5\times pen\times (m-1)}{n}{)}^{2}}$$

in which $MSE$ is the mean square error, $m$ is LBF number, $n$ means observations training dataset number, $pen$ defines a penalty factor, recommended by Friedman and Jekabsons, and it is in the range of Refs.^69,70^.

### Wavelet theory

In order to reach an appropriate analysis of non-stationary signals, wavelet transform (WT) is operated as a novel and efficient method. It was owing to the fact that this method is more flexible than Fourier transform, with it bringing about flexibility between the time scale and frequency^71^. Likewise, the new method has the advantage of analyzing signals, albeit at diverse degrees of the time scale. To put it simply, a wavelet works as a time function with fluctuations, and its energy is restricted to a fixed span of time. Provided that $\varphi$ is considered to detect the mother wavelet, the continuous wavelet transform (CWT) is defined by the equation mentioned below^72,73^:

22 $$\omega \left(b,c\right)=\int f(t)\times \left(\frac{1}{\sqrt{b}}\right)\times \varphi \left(t-\frac{c}{b}\right)dt,$$

where *b* factor is a scale and depicts the stretch or duration of the wavelet. *c* factor is a transfer parameter supplying the required time concentration and defining the point of wavelet on the time pivot. In addition, the discrete type of wavelet transform (DWT) is highly likely to be utilized in order for analyzing the time series due to the fact that time discrete series are conventional in hydro-climatological works. At any spot in the signal (*b*) and for any scale value (*c*), the coefficients of wavelet are measurable by the equation mentioned below:

23 $$ \varphi_{b,c} \left( t \right) = \frac{1}{\sqrt b }\varphi \left| {\frac{t - c}{b}} \right|. $$

Regarding DWT, these transform and scale factors are disconnected as,

24 $$ b = 2^{k} , c = 2^{k} l. $$

In fact, *k* and *l* are integers. By changing *b* and *c* in relation, the following equations can be obtained (25):

25 $$ \varphi_{k,l} \left( t \right) = 2^{ - k/2} \varphi \left[ {2^{ - k} t - l} \right]. $$

Therefore, the wavelet function is discrete wavelet. The DWT can be:

26 $$ \omega \left( {b,c} \right) = 2^{ - k/2} \smallint f\left( t \right) \times \varphi (2^{ - k} t - l)dt. $$

### Proposed ANFIS-ADEPSO

**Figure 2:**
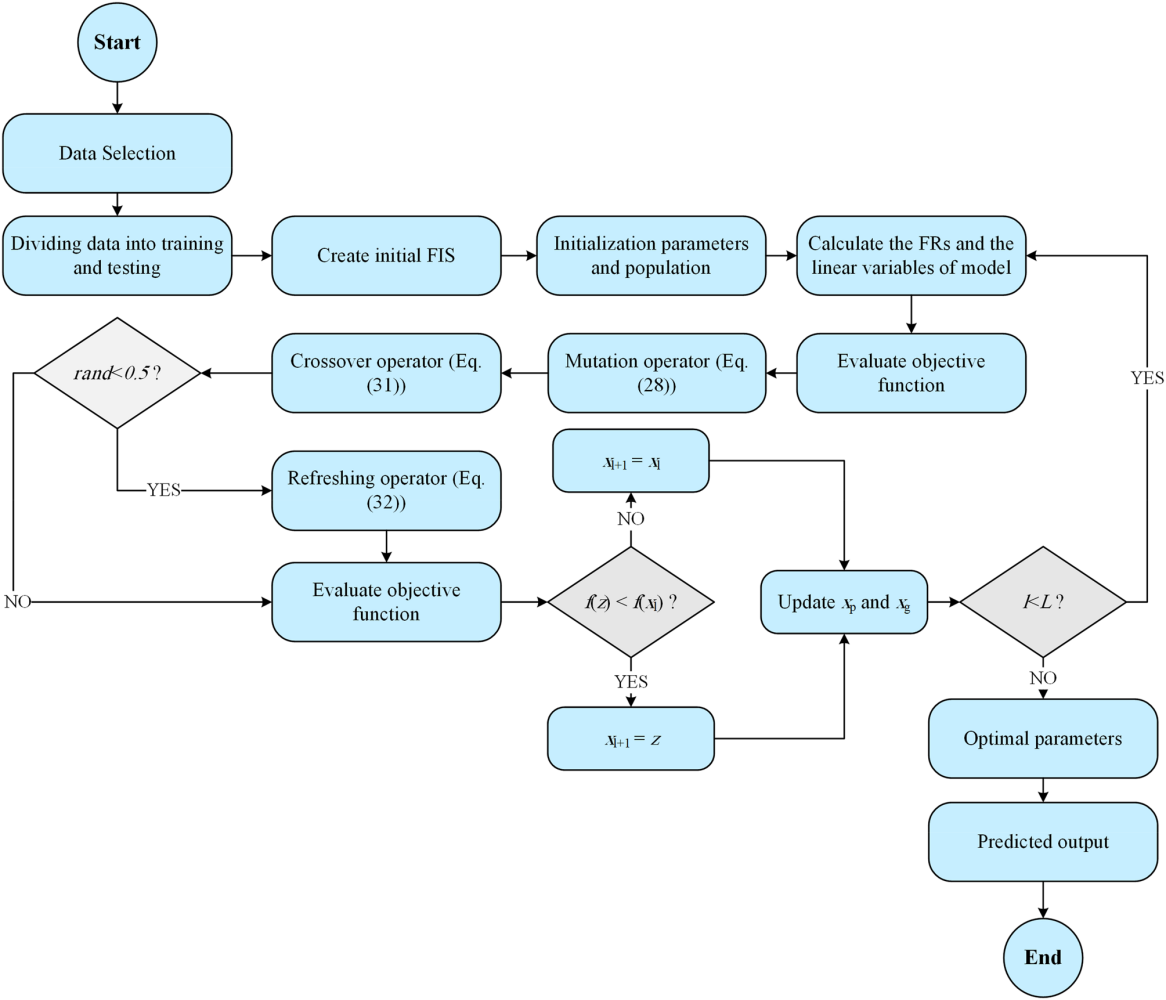
Flowchart of the proposed ANFIS-A-DEPSO model.

ANFIS model uses a classical optimization method to minimize the difference between the target and estimated outputs. The optimization method combines least squares solver (LSS) and gradient descent (GD) methods. The optimal MFs of input parameters and coefficients of the linear relation of FRs is determined by the hybrid optimization method during the training stage. One of the most severe critiques concerning the classical optimization methods is getting stuck in local solutions^36^, where employing metaheuristic optimization methods such as A-DEPSO can be a helpful choice^74^. The flowchart of the A-DEPSO algorithm coupled with the ANFIS model is displayed in Fig. 2 and expressed in the following section.

#### Adaptive hybrid of DE and PSO (A-DEPSO)

In this study, the adaptive hybrid of DE and PSO (A-DEPSO) introduced by Ref.^74^ is used to determine the ANFIS model's decision parameters, in which mutation, crossover, refreshing, and selection operators are main operators. The proposed A-DEPSO algorithm is described in the following sections.

##### Mutation in A-DEPSO

Generally, a mutation operator can promote the efficiency of an optimization method^75,76^. The A-DEPSO algorithm takes advantage of a powerful mutation operator for increasing the local and global searchability. The proposed mutant vector (${XDE}_{l\text{,}j}$) is generated by the mutant vector of DE (Eq. 27) and the vector created by the PSO algorithm (Eq. 29), which is formulated as,

27 $$ XDE_{{l{,}j}} = x_{l,j} + G.\left( {xp_{{l{,}j}} - x_{l,j} } \right) + G.\left( {x_{a1} - x_{a2} } \right), $$

28 $$ V_{{l{,}j}} = w.V_{{l{,}j}} + c_{1} .rand_{1} .\left( {xp_{{l{,}j}} - x_{l,j} } \right) + c_{2} .rand_{2} .\left( {x_{g} - x_{l,j} } \right), $$

29 $$ XPSO_{{l{,}j}} = x_{l,j} + V_{{l{,}j}} , $$

30 $$ X_{new} = \rho {{ \times }}XPSO_{{l{,}j}} { + (1} - \rho ){{ \times }}XDE_{{l{,}j}} , $$

31 $$ G = \sin \left( {\beta \times \pi \times \left( \frac{l}{L} \right)} \right) \times \exp \left( { - \frac{l}{L}} \right) \times \left( {0.5 + 0.15 \times randn} \right), $$

32 $$ w = \delta \times \exp \left( { - \frac{l}{L}} \right), $$

where $\beta$ and $\delta$ denote two constant number, $G$ denotes an adaptive parameter for scaling the differential vectors, $\rho$ is a random number in the range of [0, 1], ${c}_{1}$ and ${c}_{2}$ denote two constant numbers in which their values are equal to each other and equal to 1.5. ${rand}_{1}$ and ${rand}_{2}$ denote two random number in the range of [0, 1], $w$ denotes an inertial factor to control the velocities of particles. $randn$ denotes a random number with normal distribution. $l$ and $L$ denote the number of iteration and the maximum number of iterations, correspondingly. ${xp}_{l\text{,}j}$ and ${x}_{g}$ are the personal best of solution $j$ and best-so-far solution, correspondingly.

##### Crossover in A-DEPSO

The A-DEPSO uses a new binomial crossover (BC) to boost the population diversity. The BC merges three vectors, comprising the vector ${X}_{new}$, ${x}_{g}$, and the current solution (${x}_{l,j}$) by utilizing an adaptation rate parameter (${A}_{r}$) in which creating the crossover vector (${z}_{i}$) is done by the following equation:

33 $${z}_{i}=\left\{\begin{array}{l}{X}_{new,i} \quad \left( if {p}_{a}<{A}_{r} \; and \; {p}_{b}<0\text{.}5\right) \; or \; i= {i}_{rand } \\ {x}_{j,i} \quad  \left(if {p}_{a}<{A}_{r} \;  and \; {p}_{b}>0\text{.}5\right) \; or \; i= {i}_{rand} \\ {x}_{g,i} \quad  if \; {p}_{a}>{A}_{r},\end{array}\right.$$

34 $${A}_{r}={m}_{cr}+0.1\times randn,$$

where ${A}_{r}$ denote the adaptive rate for the BC, which is expressed as Eq. (34). ${p}_{a}$ and ${p}_{b}$ denote two random parameters in the range of [0,1], ${i}_{rand}$ denote a random integer number in the range of [1, *D*]. ${m}_{cr}$ is equal to 0.5 in the first iteration and its value can be changed based on the relationship suggested by Ref.^77^, is defined as,

35 $${m}_{cr}=\left(1-\mu \right)\times {m}_{cr}+\mu \times mean\left({S}_{Ar}\right),$$

where $\mu$ is equal to 0.1. ${S}_{Ar}$ denotes all successful ${A}_{r}$ during whole iterations. According to Eq. (35), the A-DEPSO determines the best amount for ${A}_{r}$ at each iteration and assists it to implement an appropriate search in the solution space.

##### Refreshing operator in A-DEPSO

Refreshing operator (RO) is added to the A-DEPSO for enhancing the convergence speed. The RO can create the vector (${z}_{i}$) based on the solution (${z}_{i}$) generated by the BC and two solutions ${x}_{1}$ and ${x}_{2}$. In fact, two solutions ${x}_{1}$ and ${x}_{2}$ are to promote the exploitation capability in the A-DEPSO. Thus, the RO is formulated as,

36 $$z=\left\{\begin{array}{l}{x}_{1 } \; if \; rand<LC \; and \; rand<0.5\\ {x}_{2} \; if \; rand<LC \; and \; rand>0.5\\ z \; if \; rand>LC\end{array}\right.$$

in which

37 $${x}_{1}=z+\sigma .\left(2.randn.{x}_{g}- {x}_{l\text{,}j}\right),$$

38 $${x}_{2}={x}_{g}+\sigma \times \left({x}_{a1}- {x}_{a2}\right),$$

where $LC$ denote a logistic chaotic map^78,79^ ($LC=4.LC.(1-LC)$) and its initial value is 0.7. The LC is applied to increase the random behavior of A-DEPSO and avoid from local solutions.

##### Selection operator in A-DEPSO

A-DEPSO uses the selection operator (SO) to determine whether the solution $z$ is better than the current solution (${x}_{l\text{,}j}$) or not. Based on the SO, the solution in the next iteration (${x}_{l+1\text{,}j}$) can be formulated as,

39 $${x}_{l+1\text{,}j}=\left\{\begin{array}{ll}z \quad if \; f(z) >f({x}_{l\text{,}j}) \\ {x}_{l\text{,}j} \quad otherwise.\end{array}\right.$$

### Performance evaluation

This section introduces seven statistical metrics for assessing the efficiency of five ML models, including Root mean square error (RMSE)^39^, Correlation coefficient (R)^6^, Mean absolute error (MAE)^80^, Relative absolute error (RAE)^80^, Willmott's agreement Index (IA)^81^, Legate and McCabe's Index ($E$)^82^, and mean absolute percentage error (MAPE)^83^, which are formulated as,

40 $$ RMSE = \left( {\frac{1}{M}\sum\limits_{{k = 1}}^{M} {\left( {EC_{{P,k}}  - EC_{{o,k}} } \right)^{2} } } \right)^{{0.5}} , $$

41 $$R=\frac{\sum_{k=1}^{M}\left({EC}_{P,k}-\overline{{EC }_{P}}\right).({EC}_{o,k}-\overline{{EC }_{o}})}{\sqrt{\sum_{k=1}^{M}{({EC}_{P,k}-\overline{{EC }_{P}})}^{2}\sum_{k=1}^{N}{({EC}_{o,k}-\overline{{EC }_{o}})}^{2}}},$$

42 $$RAE=\frac{{\sum }_{k=1}^{M}\left|{EC}_{P,k}-{EC}_{o,k}\right|}{{\sum }_{k=1}^{M}\left|{EC}_{P,k}-\overline{{EC }_{o}}\right|},$$

43 $$ MAE = \left( {\frac{1}{M}} \right)\sum\limits_{{i = 1}}^{M} {\left| {EC_{{P,k}}  - EC_{{o,k}} } \right|,}  $$

44 $$ MAPE\left( \%  \right) = \left( {\frac{{100}}{M}} \right)\sum\limits_{{k = 1}}^{M} {\left| {\frac{{EC_{{P,k}}  - EC_{{o,k}} }}{{EC_{{o,k}} }}} \right|,}  $$

45 $$IA=1-\frac{\sum_{k}^{M}{\left({EC}_{P,k}-{EC}_{o,k}\right)}^{2}}{\sum_{k=1}^{M}{\left(\left|\left({EC}_{P,k}-\overline{{EC }_{P}}\right)\right|+\left|\left({EC}_{o,k}-\overline{{EC }_{o,k}}\right)\right|\right)}^{2}}, 0<IA\le 1,$$

46 $$E=1-\frac{{\sum }_{k=1}^{M}\left|{EC}_{o,k}-{EC}_{P,k}\right|}{{\sum }_{k=1}^{M}\left|{EC}_{o,k}-\overline{{EC }_{P}}\right|},$$

where ${EC}_{o,k}$ denotes the observed EC value, ${EC}_{P,k}$ denotes the predicted EC value, $\overline{{EC }_{P}}$ and $\overline{{EC }_{o,k}}$ denote average values of observed and predicted EC, respectively, and *M* denotes the number of data samples. In addition, if the RMSE, MAE, RAE and MAPE are near to 0 and R, E and I_A_ are near to 1, the model presents better efficiency.

Since specifying the best model according to seven metrics is a difficult issue, the multi-index criterion (PI) (Wang et al., 2018) is used in this study to make an easy decision for selecting the best model. The formula of PI is defined as,

47 $$PI=\frac{1}{7}.\left(\frac{{R}_{min}}{R}+\frac{RMSE}{{RMSE}_{max}}+\frac{MAE}{{MAE}_{max}}+\frac{RAE}{{RAE}_{max}}+\frac{MAEP}{{MAEP}_{max}}+\frac{{E}_{min}}{E}+\frac{{IA}_{min}}{IA}\right),$$

where ${R}_{min}$, ${E}_{min}$, and ${IA}_{min}$ are the minimum values of $R$, $E$, and $IA$ achieved by all ML methods. Also, ${RMSE}_{max}$, ${MAE}_{max}$, and ${MAEP}_{max}$ are the maximum values of $RMSE$, $MAE$, and $MAEP$ obtained by all ML models.

## Study area

**Figure 3:**
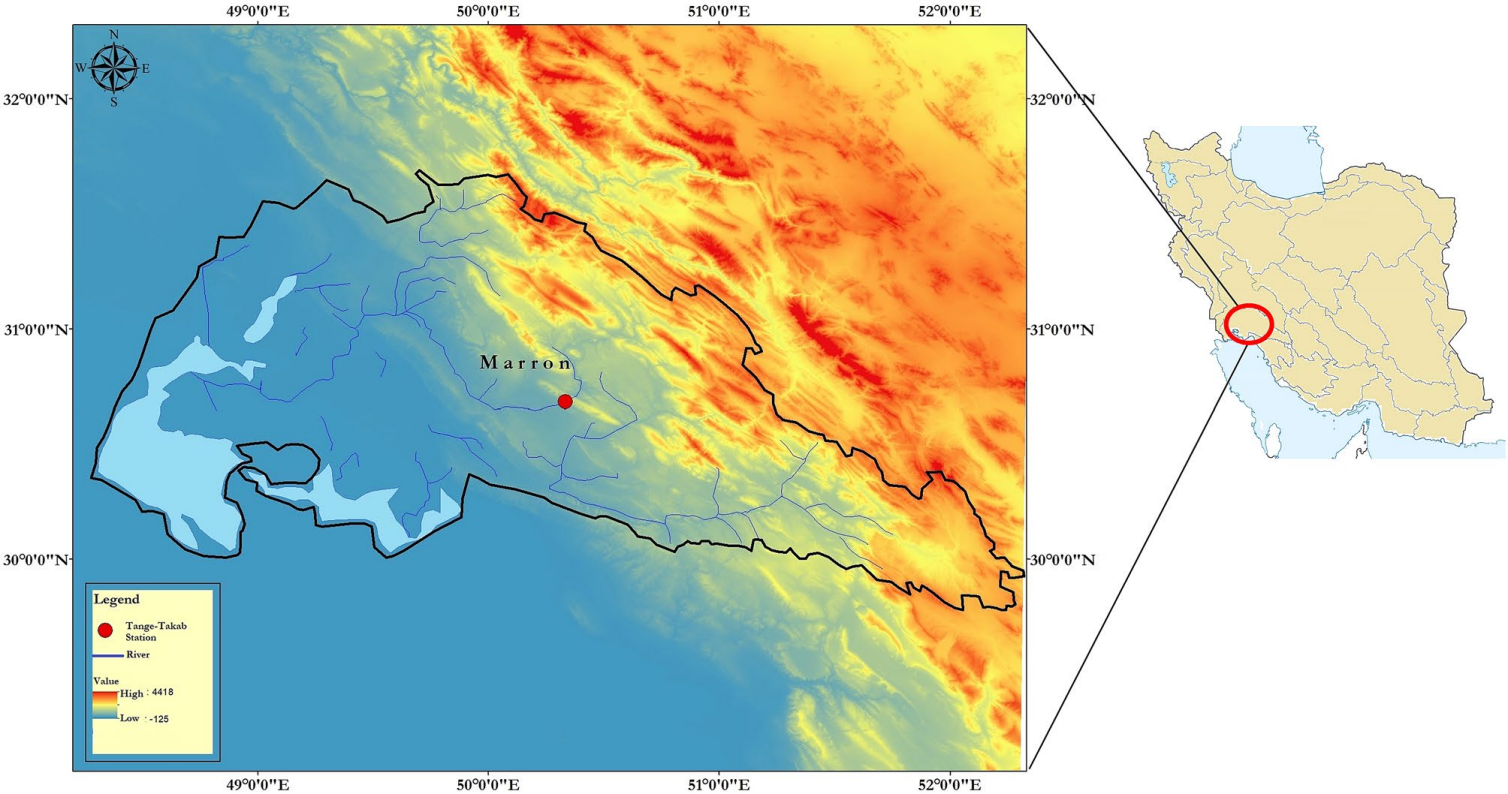
Location of Tange-Takab station.

In this study, the parameters selected on a monthly basis consisting of discharge and electrical conductivity, which is originated from the Tange-Takab gauging station (Longitude 50° 20′ 02″, Latitude 30° 41′ 09″, and 280 m from mean sea level) and located on the Maroon River of Khuzestan province, Iran. The exact location of the Tange-Takab gauging station is illustrated in Fig. 3. Needless to say, this river, with a drainage area of 6824 km^2^ and almost 310 km long, has a profound impact on supplying drinking water, irrigation and recreation for Iranians, in particular southeastern regions' residents in Iran. More specifically, this area, the Maroon basin, witnesses almost the average of 24 °C temperature and 350.04 mm precipitation annually.

### Pre-processing and selecting the best combination

**Figure 4:**
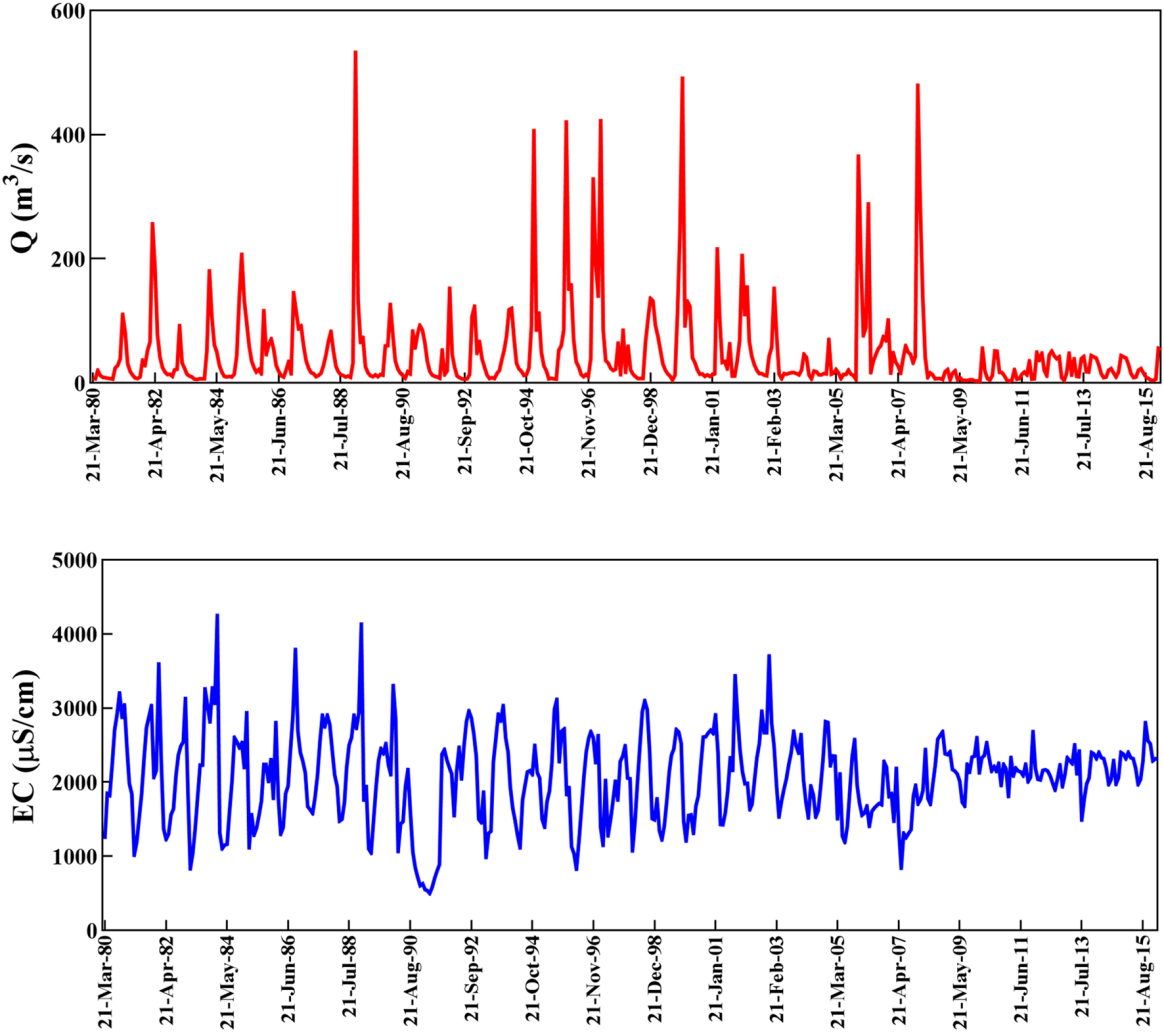
Original time series of Q (input) (upper graph) and EC (target) (lower graph) for all dataset.

The data collected in a span of 36 years (21-March-1980–16-Feb-2016, 432 months) is to simulate EC on a monthly basis through ML models. The given data provided on a monthly time step are segregate into two distinct sections, namely train and test, as 70% (302-month) and 30% (130-month) of whole data is dedicated to training and testing set respectively. As can be observed, Fig. 4 (upper) depicts independent variables, being the time series of Q and Fig. 4 (lower) also illustrates the EC time series being considered a purpose in training and testing periods. Table 1 provides a classification of various statistical criteria such as minimum (MIN), maximum (MAX), average (AVG), range, standard deviation (SD), skewness (S), kurtosis (K), and autocorrelation coefficients (AC) for training, testing, and all data points. From what has been mentioned in the Table, it is clear that the S and K amounts of EC for both training and testing datasets have the same range of ([− 2, 2]), whereas the S range ([3.469, 6.49]) and K range ([15.4, 48.81]) of Q reveals the distribution of discharge time series is away from normal distribution.

**Table 1:** Statistic information of dataset utilized in train and test.

Mode	Statistics	Q (m^3^/s)	Ec (μS/cm)
Train	MIN	4.9	503
	MAX	532	4250
	Range	527.1	3747
	AVG	53.09	2049
	SD	73.42	655.3
	S	3.469	0.1256
	K	15.14	− 0.01149
Test	MIN	3.06	845
	MAX	479	2800
	Range	475.9	1955
	AVG	29.6	2134
	SD	54.17	321.4
	S	6.49	− 1.205
	K	48.81	2.547
AC	R_1_	0.439	0.690
	R_2_	0.216	0.441
	R_3_	0.099	0.197
	R_4_	− 0.045	− 0.039

The step of choosing the optimum combination of input variables in time series concerning forecasting models by ML models is considered a significant stage, in which the consecutive time series lagged data is influential to a great extent^84–86^. There is not any criterion to specify the number of lags; however, the auto-correlation function (ACF), partial auto-correlation function (PACF) and cross correlation (CC) statistical methods are considered to detect the input combination on hydrological models^6,87^.

**Figure 5:**
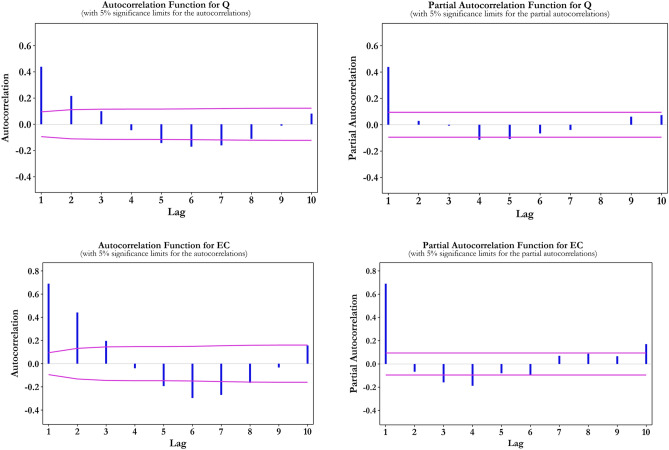
ACF and PACF of input and target datasets.

In Fig. 5 the AF is operated to estimate the effective input parameter. As can be observed, the AC of 1-month and 2-month lagged signals has a more significant influence (more than 55%) on the original input datasets ($Q_{t}$) and $EC_{t}$ in comparison with the lagged times ($Q_{t - 3} {, }Q_{t - 4} {,} \ldots {,; }EC_{t - 5}$). In addition, based on the PCFA, the 1-month lagged signal for $Q_{t}$ and 1-month to 4-month lagged signals for $EC_{t}$ can be considered.

**Figure 6:**
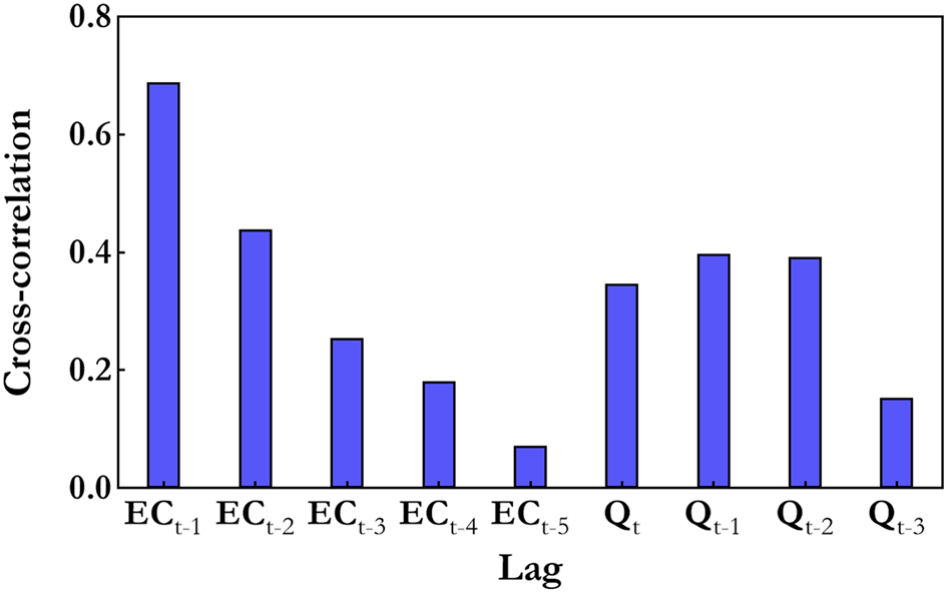
Cross correlation between input and target dataset.

The Fig. 6 reveals the high correlation belongs to the input single (Q_t_) at the current time, the first two time-lagged signals (${Q}_{t-1}\text{, }{Q}_{t-2}$) and first four time-lagged signals of $EC_{t}$($E{C}_{t-1}\text{, }E{C}_{t-2},E{C}_{t-3},E{C}_{t-4}$), which has more significant effect on creating a predictive model compared to the $Q_{t - 3}$ and $E{C}_{t-5}$. To add to it, comparing the cross-correlation values between target signals ($E{C}_{t}$) and the input signals proves that the $E{C}_{t-1}\text{, }E{C}_{t-2}$, with greater correlation coefficients, by 0.68 and 0.45 respectively, play an important part in predicting the WQ parameter of the target. As a result, by analyzing ACF and CC, it is clear that the lagged t of up to 2 and 4 months for the current month predicting of $EC_{t}$ were accepted. Then, in order to determine the best input patterns amongst all available and possible patterns, one of the best subset regression analyses in this research was assessed. Simply put, to choose the optimal input pattern for each WQ target, four distinct criteria, namely of R^2^, adjusted R^2^, Mallows (${C}_{P}$)^88^, and Amemiya prediction criterion (PC)^89^ are used. In the following $C_{P}^{{}}$ and PC are defined^90^:

48 $${C}_{p}=\frac{RS{S}_{i}}{MS{E}_{m}}+2i-N,m>i,$$

49 $$PC=\left(\frac{n+i}{n-i}\right)\left(1-\left({R}^{2}\right)\right).$$

In which $MS{E}_{m}$ expresses mean squared error, *i* is predictors' number, $RSS_{i}$ is considered the residual sum of squares and *N* is the historical dataset's number. With regard to Table 2, in which the best subset regression analysis's result $EC_{t}$ is classified, the analysis $EC_{t}$ was evaluated to selected four the most appropriate pattern, the optimum input data for predictive models, based on the best result of factors such as R^2^ ([56.90 57.00%]), $C_{P}^{{}}$([4.74 8.00]) and PC ([0.441 0.444]). It is true to say; this method is unlikely to ensure alone the accuracy of the most suitable input combinations. In turn, taking other statistical conditions, including the Pearson correlation between basic input parameters and the purpose parameters and multicollinearity interaction analysis between inputs, into account is an essential matter to raise the certainty of the combination selection. Admittedly, the current Q and time-lagged EC time series affect considerably the input combination of purpose signal. Hence, in order to predict the current $EC_{t}$ on a monthly basis, four input mixtures were separately provided with the purpose of enhancing ML based on predictive models categorized in the form of boldface in Table 2.

**Table 2:** Best subset analysis to optimally chosen the input combination of EC in ML models.

	Combinations	R^2^%	R^2-Ajd^%	C_p_	PC
1	EC_t−1_	0.474	0.473	90.660	0.528
2	Q/EC_t−1_	0.550	0.547	18.365	0.455
3	Q/EC_t−4_/EC_t−1_	0.563	0.560	6.770	0.443
**4**	**Q**_**t−2**_**/Q/EC**_**t−4**_**/EC**_**t−1**_	**0.567**	**0.563**	**4.678**	**0.441**
**5**	**Q**_**t−2**_**/Q/EC**_**t−4**_**/EC**_**t−2**_**/EC**_**t−1**_	**0.569**	**0.564**	**4.741**	**0.441**
**6**	**Q**_**t−2**_**/Q**_**t−1**_**/Q/EC**_**t−4**_**/EC**_**t−2**_**/EC**_**t−1**_	**0.570**	**0.564**	**6.119**	**0.442**
**7**	**Q**_**t−2**_**/Q**_**t−1**_**/Q/EC**_**t−4**_**/EC**_**t−3**_**/EC**_**t−2**_**/EC**_**t−1**_	**0.570**	**0.563**	**8.000**	**0.444**

Significant values are in bold.

## Application results and discussion

In this paper, the A-DEPSO is developed to find optimal parameters of the ANFIS model and to enhance the convergence speed. The efficiency of the proposed method is compared with LSSVM, MARS, and GRNN models to predict the EC parameter in standalone and wavelet-complementary frameworks. In this regard, ANFIS-A-DEPSO can predict the EC, with it overcoming the demerits of the basic ANFIS algorithm by optimizing coefficients detecting membership functions. Thus, it is concomitant with more meticulous predictive outcomes. In fact, the A-DEPSO optimization method is used to extract optimal parameters of the ANFIS model and increase the precision and speed of convergence rate. Furthermore, the position of each member in the A-DEPSO algorithm indicate the amounts of consequent (${\alpha }_{1}$, ${\beta }_{1}$, ${\gamma }_{1}$, ${\alpha }_{2}$, ${\beta }_{2}$, ${\gamma }_{2}$) and membership parameters (${a}_{k}$, ${e}_{k}$, ${m}_{k}$) in the ANFIS model. The baseline parameter values were treated as the starting locations of the solutions. To validate prediction accuracy, the fitness function of root mean square error (RMSE) was used. The hybrid ANFIS model were run until the RMSE was reduced to a minimum and the methods were converged toward the best solutions. Within every update of the solution' positions, the ideal amounts of design variables were discovered.

Likewise, operating the ANFIS, LSSVM, MARS, and GRNN as striking machine learning methods were useful to confirm the predictive ability of ANFIS-A-DEPSO, which leads to the major novelty of this research. During a trial-and-error manner, the LSSVM, GRNN, and MARS gained their substantial setting parameters. The given Table 3 classifies the amounts of control parameters for these mentioned methods. It should be noted that the population size and the maximum number of iterations for the A-DEPSO algorithm are equal to 50 and 200, respectively.

**Table 3:** Control parameters of the LSSVM, MARS, and GRNN models.

Model	Parameters
LSSVM	$\lambda =4989$ and $\gamma =1265$
MARS	Number of BFs = 22
ANFIS	Number of membership functions = 5
ANFIS-ADEPSO	$\beta =50$ and $\delta =1$
GRNN	Spread value = 492

### Wavelet-ML models

As another effective model in terms of the certainty of predictive hydrological models can refer to the complementary data-intelligence models, including wavelet discrete or continuous wavelet transforms (DWT and CWT, correspondingly) as well ML model, which stems from a appropriate mother wavelet and decomposition level disintegration. It is commonly observed, two mother wavelet, namely as discrete Meyer (Damey) and Biorthogonal 6.8 (Bior 6.8) have proven their noteworthy ability in WQ predictive models, mainly because they support condensed form and are useful in producing time localization^12,15,20^. In this research, the mother wavelet (i.e., bior6.8, and dmey) was used to break up the time series. In the following, the optimal disintegration level (1) of wavelet transform for the WQ time series was formulate^6^:

50 $$ nMW = int\left[ {\log \left( N \right)} \right]. $$

**Figure 7:**
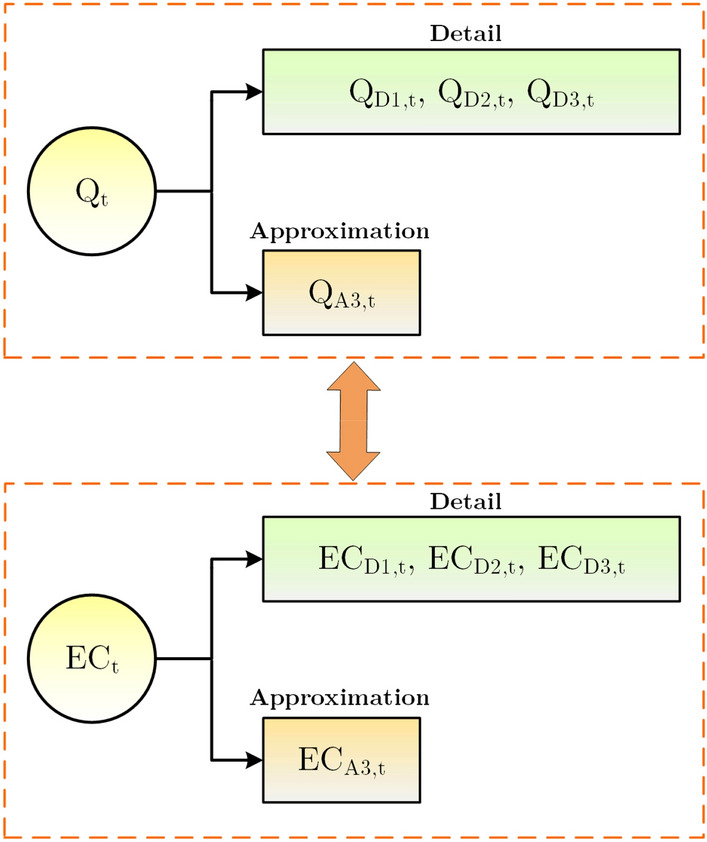
Decomposition of datasets using DWT.

In which *N* describes the dataset's number, accounting for 432. So, the figure of disintegration level will be 3. As a result, the used basic signals in the EC modeling were divided into three levels of details and approximations as Fig. 7.

**Figure 8:**
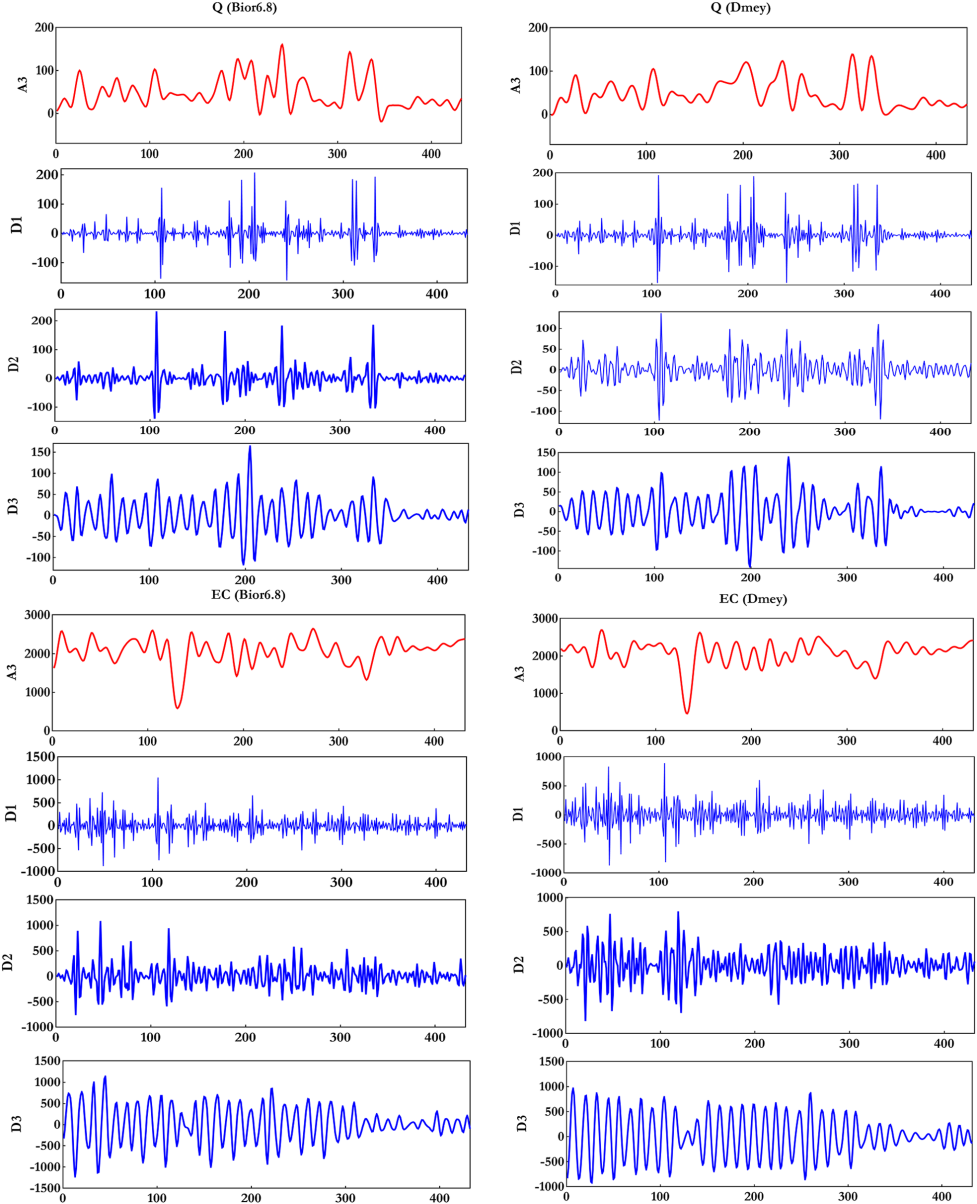
Decomposition of Q and EC datasets for two mother wavelets (Bior6.8 and Dmey).

**Figure 9:**
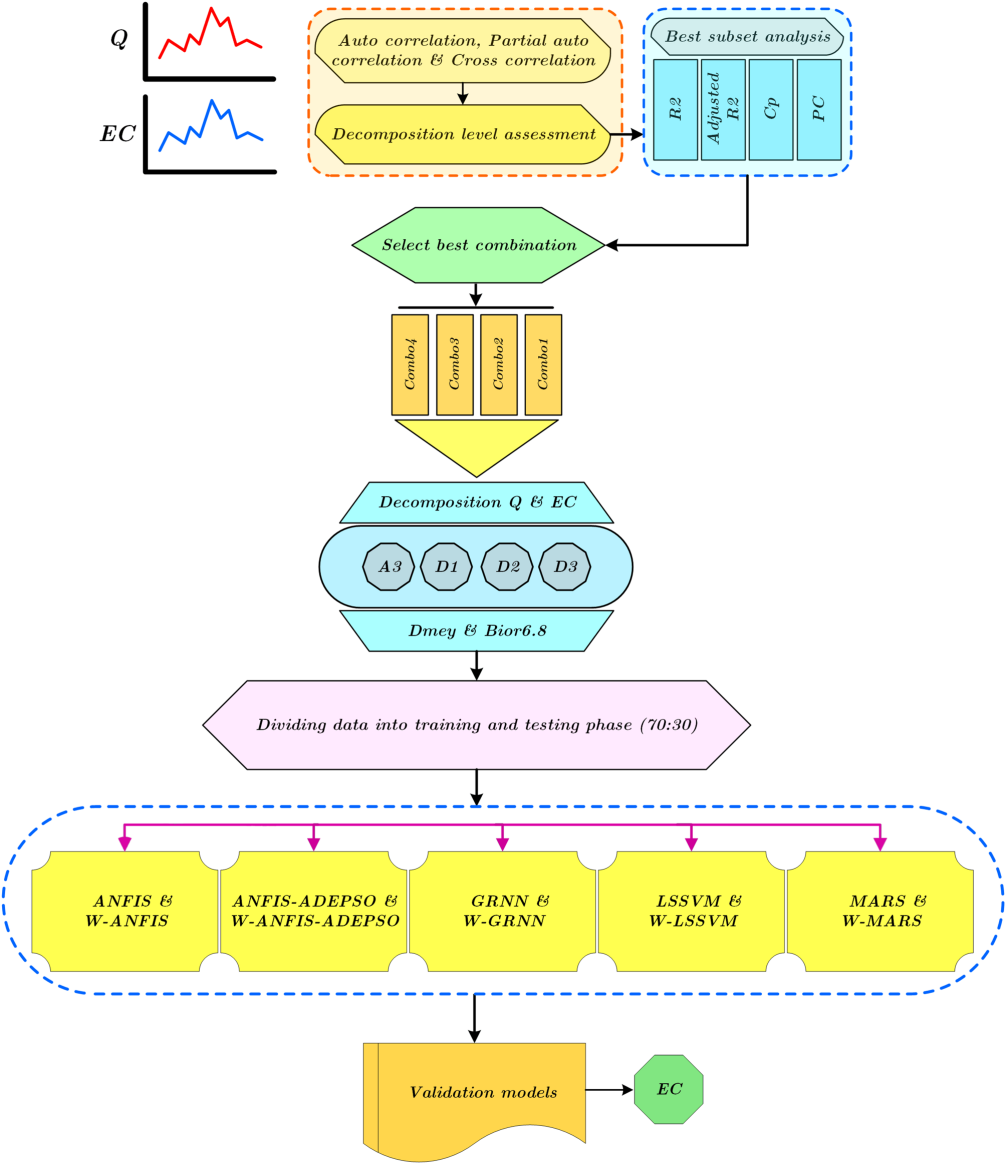
Flowchart of all ML models for forecasting EC.

In the next step, influential sub-series was collected (e.g.,$Q = A_{3} + \sum\nolimits_{i = 1}^{3} {D_{i} }$) and arranged as the input variables for supplementary five ML models based on the input combinations for the EC. Figure 8 demonstrates the details (Ds) and approximations (As) of separated signals of the EC simulation. Figure 9 displays the flowchart of ML models for forecasting EC parameters.

### Evaluate the performance of standalone ML models

In this subsection, four various combinations of input parameters given in Tables 4 and 5 evaluated the ability of five standalone ML models in forecasting the $E{C}_{t}$ for training and testing stages, respectively. Based on the previous studies^3,11,44,91^, the models having the best results in the test period show the best performance, whereby the results of the test period will be examined in order to determine the best model in this study. In fact, the outcomes of the ANFIS-A-DEPSO model, the first standalone one, is addressed. In this regard, Table 5, in which the performance of the ANFIS-A-DEPSO model to predict the $EC_{t}$ in the testing phases provided reveals Combo 3 (R = 0.672, RMSE = 275.404, MAPE = 10.956, E = 0.394, I_A_ = 0.801, and PI = 0.832) has the edge over other combinations. Likewise, four combinations are used to recognize the most appropriate combination of input parameters in MARS and ANFIS, and the best combinations for both were equal to Combo 4. In GRNN (R = 0.676, RMSE = 301.595, MAPE = 12.165, E = 0.274, I_A_ = 0.803, and PI = 0.964) and LSSVM (R = 0.620, RMSE = 284.036, MAPE = 11.843, E = 0.356, I_A_ = 0.747, and PI = 0.948), the most suitable mixture is combo 4.

**Table 4:** Statistic metrics obtained by five ML models to forecast the EC parameter in training stage.

Model	Criteria	Combination			
		Combo 1	Combo 2	Combo 3	Combo 4
ANFIS-A-DEPSO	R	**0.848**	0.845	0.846	0.844
	RMSE	**353.197**	356.313	355.442	359.698
	MAE	**243.342**	253.611	244.956	248.578
	RAE	**0.449**	0.468	0.452	0.459
	MAPE	**12.960**	14.165	13.460	12.950
	E	**0.719**	0.714	0.715	0.709
	I_A_	**0.914**	0.909	0.910	0.905
	PI	**0.977**	1.000	0.983	0.982
ANFIS	R	0.841	**0.841**	0.836	0.841
	RMSE	360.181	**360.099**	366.059	360.440
	MAE	250.811	**250.091**	257.408	252.690
	RAE	0.463	**0.461**	0.475	0.466
	MAPE	13.740	**13.631**	14.596	13.908
	E	0.708	**0.708**	0.698	0.707
	I_A_	0.908	**0.908**	0.905	0.908
	PI	0.984	**0.982**	1.000	0.987
LSSVM	R	0.825	0.826	**0.829**	0.827
	RMSE	376.503	375.911	**373.261**	375.117
	MAE	257.907	257.315	**254.815**	256.712
	RAE	0.476	0.475	**0.470**	0.474
	MAPE	14.637	14.510	**14.377**	14.506
	E	0.681	0.682	**0.686**	0.683
	I_A_	0.895	0.895	**0.897**	0.895
	PI	1.000	0.998	**0.994**	0.997
GRNN	R	0.790	0.811	**0.813**	0.804
	RMSE	417.344	398.072	**396.565**	408.553
	MAE	292.164	278.826	**277.396**	289.653
	RAE	0.539	0.514	**0.512**	0.534
	MAPE	16.998	16.151	**16.068**	16.941
	E	0.608	0.643	**0.646**	0.624
	I_A_	0.848	0.868	**0.870**	0.855
	PI	1.000	0.981	**0.979**	0.996
MARS	R	**0.844**	0.834	0.837	0.832
	RMSE	**357.725**	368.062	364.990	369.822
	MAE	**245.850**	252.034	256.522	258.810
	RAE	**0.454**	0.465	0.473	0.478
	MAPE	**13.273**	13.793	13.944	14.456
	E	**0.712**	0.695	0.700	0.692
	I_A_	**0.910**	0.903	0.905	0.902
	PI	**0.973**	0.986	0.992	1.000

Significant values are in bold.

**Table 5:** Statistic metrics obtained by five ML models to forecast the EC parameter in testing stage.

Model	Criteria	Combination			
		Combo 1	Combo 2	Combo 3	Combo 4
ANFIS-A-DEPSO	R	0.665	0.676	0.671	0.672
	RMSE	304.606	289.100	317.061	**275.404**
	MAE	224.062	211.387	241.597	**198.092**
	RAE	0.807	0.762	0.871	**0.714**
	MAPE	12.452	11.573	13.049	**10.956**
	E	0.259	0.333	0.197	**0.394**
	I_A_	0.792	0.799	0.804	0.801
	PI	0.933	0.874	0.996	**0.832**
ANFIS	R	0.626	0.620	0.650	**0.659**
	RMSE	350.233	359.110	372.765	**320.470**
	MAE	258.478	268.127	283.825	**241.088**
	RAE	0.931	0.966	1.023	**0.869**
	MAPE	13.954	14.229	14.933	**12.863**
	E	0.020	-0.030	-0.110	**0.180**
	I_A_	0.764	0.762	0.772	**0.790**
	PI	0.947	0.968	0.990	**0.887**
LSSVM	R	**0.676**	0.676	0.667	0.671
	RMSE	**301.595**	303.898	309.440	303.767
	MAE	**221.156**	223.020	225.851	221.441
	RAE	**0.797**	0.804	0.814	0.798
	MAPE	**12.165**	12.264	12.415	12.168
	E	**0.274**	0.263	0.235	0.263
	I_A_	**0.803**	0.800	0.795	0.800
	PI	**0.964**	0.975	1.000	0.972
GRNN	R	0.620	0.628	0.631	0.616
	RMSE	**284.036**	288.484	290.054	295.266
	MAE	**214.482**	221.582	222.083	223.623
	RAE	**0.773**	0.798	0.800	0.806
	MAPE	**11.843**	12.247	12.303	12.565
	E	**0.356**	0.335	0.328	0.304
	I_A_	0.747	0.750	0.750	0.724
	PI	**0.948**	0.969	0.973	1.000
MARS	R	0.630	0.632	0.621	**0.648**
	RMSE	332.071	327.517	322.743	**314.030**
	MAE	249.266	247.801	233.385	**231.351**
	RAE	0.898	0.893	0.841	**0.834**
	MAPE	13.642	13.405	12.773	**12.723**
	E	0.119	0.143	0.168	**0.213**
	I_A_	0.764	0.772	0.770	0.786
	PI	0.998	0.966	0.926	**0.890**

Significant values are in bold.

**Figure 10:**
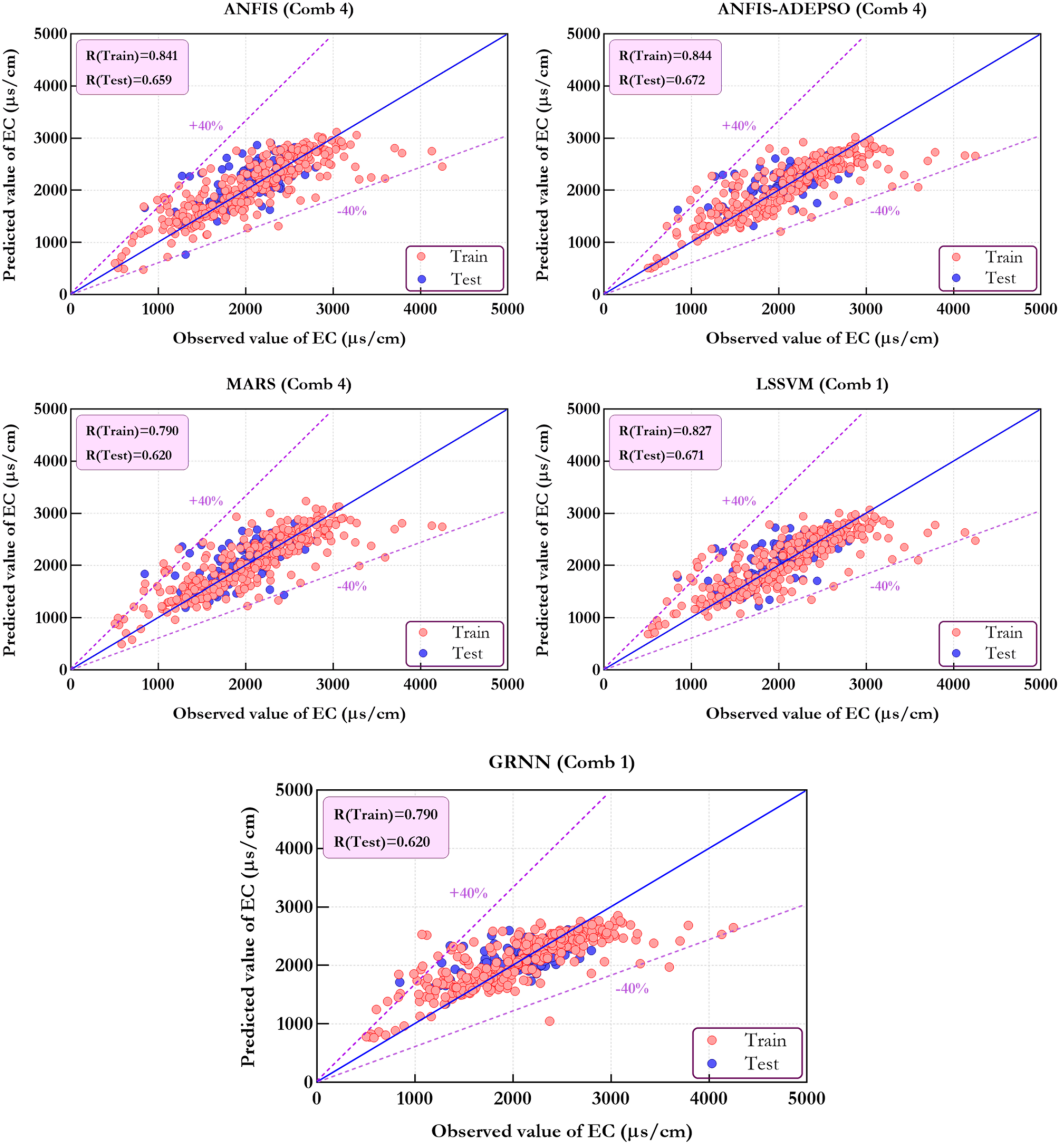
Compare estimated and measured values of EC utilizing the standalone ML models in the form of scatter plot.

The given Fig. 10 provides the data on the observed against predicted WQP amounts for training and testing phases. It is clear that the proportion of error in predicted amounts gained through two ML models accounts for ± 40%. Therefore, five standalone ML models are not appropriate to predict the EC.

**Figure 11:**
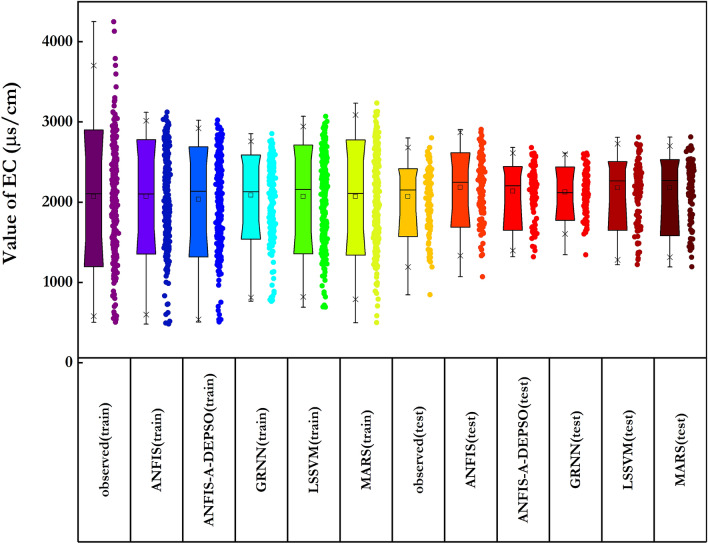
Assessing the distribution of estimated and measured values of EC obtained by all ML models.

Figure 11 illustrates the distribution of predicted and measured amounts of the EC, which is obtained through the ANFIS-A-DEPSO, ANFIS, LSSVM, MARS and GRNN models for all datasets. More specifically, it is notable that the disparity between the predicted and measured amounts of the EC, resulting in the five standalone ML models, are not able to predict the EC accurately.

### Evaluate the performance of wavelet-based ML models

In this study, the W-ANFIS-A-DEPSO, W-ANFIS, W-LSSVM, W-GRNN, and W-MARS models are enhanced to boost five standalone ML models' accuracy (i.e., ANFIS-A-DEPSO, ANFIS, MARS, GRNN, and LSSVM). As mentioned before, decomposing the time series of EC is implemented by two mother wavelets (i.e., bior6.8 and dmey). Four mixtures of input variables are used to address the ability of the W-ML models with diverse mother wavelets. The given Table 6 provides data on optimal parameters of the all-wavelet-based models.

**Table 6:** Control parameters of the LSSVM, MARS, and GRNN models.

Model	Parameters
LSSVM	$\lambda =1992$ and $\gamma =1346$
MARS	Number of BFs = 20
ANFIS	Number of membership functions = 5
ANFIS-ADEPSO	$\beta =50$ and $\delta =1$
GRNN	Spread value = 380

The comparison of the W-ANFIS-A-DEPSO models’ prediction certainty towards two mother wavelets and all combinations are reported on Table 7, which is illustrated the W-ANFIS-A-DEPSO model with mother wavelets Dmey and Bior6.8, as the best combination, is Comb 4(R = 0.990, RMSE = 51.193, MAPE = 2.143, E = 0.979, and PI = 0.480) and Comb 1 (R = 0.988, RMSE = 54.064, MAPE = 13.5676, E = 0.977, and PI = 0.518) correspondingly for $EC_{t}$ prediction in testing phase. The outcomes reveal Dmey has the most appropriate performance comparison with the Bior6.8 for W-ANFIS-A-DEPSO, owing to the fact that it has more suitable accuracy in comparison by the Bior6.8 mother wavelet.

**Table 7:** Statistic metrics obtained by W-ANFIS-A-DEPSO model to forecast the EC parameter for all combinations.

Model	Criteria	Combination			
		Combo 1	Combo 2	Combo 3	Combo 4
Wavelet-Demy Test	R	0.866	0.960	0.962	**0.990**
	RMSE	221.322	99.818	96.881	**51.193**
	MAE	173.394	78.444	76.877	**41.870**
	RAE	0.625	0.283	0.277	**0.151**
	MAPE	9.053	4.016	4.053	**2.143**
	E	0.609	0.920	0.925	**0.979**
	I_A_	0.911	0.979	0.981	**0.995**
	PI	1.000	0.613	0.608	**0.480**
Wavelet-Bior Test	R	0.857	0.959	0.955	**0.988**
	RMSE	192.071	101.203	106.158	**54.064**
	MAE	157.716	81.614	84.647	**43.549**
	RAE	0.568	0.294	0.305	**0.157**
	MAPE	7.919	4.222	4.395	**2.222**
	E	0.705	0.918	0.910	**0.977**
	I_A_	0.914	0.978	0.977	**0.994**
	PI	1.000	0.67032	0.684	**0.518**
Wavelet-Demy Train	R	0.947	0.979	0.978	0.992
	RMSE	213.432	134.953	140.161	83.955
	MAE	164.758	102.836	104.114	65.822
	RAE	0.304	0.190	0.192	0.121
	MAPE	9.018	5.611	5.482	3.607
	E	0.897	0.959	0.956	0.984
	I_A_	0.972	0.989	0.989	0.996
	PI	1.000	0.770	0.774	0.633
Wavelet-Bior Train	R	0.947	0.979	0.978	0.994
	RMSE	213.683	134.877	138.033	75.835
	MAE	155.866	104.022	107.249	59.092
	RAE	0.288	0.192	0.198	0.109
	MAPE	8.512	5.592	5.719	3.245
	E	0.897	0.959	0.957	0.987
	I_A_	0.972	0.989	0.989	0.997
	PI	1.000	0.787	0.797	0.619

Significant values are in bold.

Concerning the W-ANFIS model, it is true to say the best combination is equivalent to all mother wavelets and Comb 4 (Table 8). In addition, the outcomes of various mother wavelets for the best combination are obtained as, W-ANFIS -Dmey (Combo4: R = 0.985, RMSE = 60.295, MAPE = 2.484, E = 0.971, and PI = 0.517), and W-ANFIS -Bior6.8 (Combo4: R = 0.984, RMSE = 64.090, MAPE = 2.543, E = 0.967, and PI = 0.539). From what has been gained, it is readily apparent that the best mother wavelet is Dmey for W-ANFIS, thanks to higher accuracy than others.

**Table 8:** Statistic metrics obtained by W-ANFIS model to forecast the EC parameter for all combinations.

Model	Criteria	Combination			
		Combo 1	Combo 2	Combo 3	Combo 4
Wavelet-Demy Test	R	0.846	0.956	0.933	**0.985**
	RMSE	210.490	104.453	128.754	**60.295**
	MAE	157.363	82.587	96.540	**46.328**
	RAE	0.567	0.298	0.348	**0.167**
	MAPE	8.402	4.219	5.091	**2.484**
	E	0.646	0.913	0.868	**0.971**
	I_A_	0.916	0.977	0.963	**0.993**
	PI	1.000	0.654	0.721	**0.517**
Wavelet-Bior Test	R	0.835	0.956	0.934	**0.984**
	RMSE	199.554	104.421	128.283	**64.090**
	MAE	153.389	82.565	92.608	**50.278**
	RAE	0.553	0.298	0.334	**0.181**
	MAPE	7.907	4.218	4.970	**2.543**
	E	0.682	0.913	0.869	**0.967**
	I_A_	0.912	0.977	0.965	**0.992**
	PI	1.000	0.670	0.729	**0.539**
Wavelet-Demy Train	R	0.939	0.976	0.978	0.986
	RMSE	228.978	146.388	138.552	112.499
	MAE	170.889	111.805	105.004	67.705
	RAE	0.315	0.206	0.194	0.125
	MAPE	9.267	5.949	5.644	3.815
	E	0.882	0.952	0.957	0.971
	I_A_	0.968	0.987	0.989	0.993
	PI	1.000	0.780	0.758	0.647
Wavelet-Bior Train	R	0.925	0.976	0.978	0.994
	RMSE	253.480	146.388	139.832	75.792
	MAE	183.088	111.803	107.431	60.205
	RAE	0.338	0.206	0.198	0.111
	MAPE	9.870	5.949	5.726	3.343
	E	0.855	0.952	0.956	0.987
	I_A_	0.960	0.987	0.989	0.997
	PI	1.000	0.746	0.731	0.579

Significant values are in bold.

In the case of W-LSSVM, based on Table 9, the best combination for two mother wavelets is Combo4. The results of Bior6.8 and Dmey mother wavelets for the best combination are: W-LSSVM -Dmey (R = 0.984, RMSE = 64.727, MAPE = 2.638, E = 0.967, and PI = 0.599), and W- LSSVM -Bior6.8 (R = 0.985, RMSE = 62.553, MAPE = 2.564, E = 0.969, and PI = 0.572). Accordingly, the results reveal that the best mother wavelet for the W- LSSVM is Bior6.8, which has a higher precision compared with the Dmey.

**Table 9:** Statistic metrics obtained by W-LSSVM model to forecast the EC parameter for all combinations.

Model	Criteria	Combination			
		Combo 1	Combo 2	Combo 3	Combo 4
Wavelet-Demy Test	R	0.887	0.956	0.952	**0.984**
	RMSE	166.303	104.268	108.997	**64.727**
	MAE	133.497	77.533	82.778	**51.466**
	RAE	0.481	0.279	0.298	**0.185**
	MAPE	7.046	4.033	4.398	**2.638**
	E	0.779	0.913	0.905	**0.967**
	I_A_	0.941	0.978	0.975	**0.992**
	PI	1.000	0.729	0.754	**0.599**
Wavelet-Bior Tesr	R	0.863	0.959	0.953	**0.985**
	RMSE	181.865	100.140	107.135	**62.553**
	MAE	137.126	75.682	81.766	**50.104**
	RAE	0.494	0.273	0.295	**0.181**
	MAPE	7.088	3.914	4.332	**2.564**
	E	0.736	0.920	0.908	**0.969**
	I_A_	0.927	0.979	0.976	**0.992**
	PI	1.000	0.693	0.723	**0.572**
Wavelet-Demy Train	R	0.938	0.980	0.980	0.993
	RMSE	231.397	132.822	131.547	78.506
	MAE	174.007	100.098	99.820	61.028
	RAE	0.321	0.185	0.184	0.113
	MAPE	9.489	5.303	5.246	3.303
	E	0.879	0.960	0.961	0.986
	I_A_	0.966	0.990	0.990	0.996
	PI	1.000	0.733	0.731	0.599
Wavelet-Bior Train	R	0.931	0.977	0.979	0.993
	RMSE	243.767	141.328	135.022	79.378
	MAE	175.774	106.906	102.476	61.849
	RAE	0.324	0.197	0.189	0.114
	MAPE	9.398	5.666	5.385	3.361
	E	0.866	0.955	0.959	0.986
	I_A_	0.962	0.988	0.989	0.996
	PI	1.000	0.747	0.731	0.596

Significant values are in bold.

According to Table 10, in which W-MARS model’s outcomes with mother wavelets Dmey and bior6.8 is reported, proves that the best combination considered for Dmey and Bior6.8 are Comb 4 (R = 0.977, RMSE = 77.944, MAPE = 3.358, E = 0.951, and PI = 0.612) and Comb 4 (R = 0.978, RMSE = 77.937, MAPE = 3.337, E = 0.951, and PI = 0.616) correspondingly. Hence, the Bior6.8, as the best mother wavelet has better performance and certainty compared to the Dmey.

**Table 10:** Statistic metrics obtained by W-MARS model to forecast the EC parameter for all combinations.

Model	Criteria	Combination			
		Combo 1	Combo 2	Combo 3	Combo 4
Wavelet-Demy Test	R	0.846	0.957	0.958	**0.977**
	RMSE	194.178	104.777	102.849	**77.944**
	MAE	141.963	81.975	80.046	**64.589**
	RAE	0.512	0.295	0.288	**0.233**
	MAPE	7.543	4.213	4.138	**3.358**
	E	0.699	0.912	0.916	**0.951**
	I_A_	0.914	0.975	0.976	**0.987**
	PI	1.000	0.691	0.684	**0.612**
Wavelet-Bior Test	R	0.853	0.943	0.942	**0.978**
	RMSE	187.507	123.239	121.978	**77.937**
	MAE	142.228	97.601	93.782	**63.880**
	RAE	0.512	0.352	0.338	**0.230**
	MAPE	7.500	5.102	5.059	**3.337**
	E	0.719	0.879	0.881	**0.951**
	I_A_	0.910	0.963	0.965	**0.987**
	PI	1.000	0.768	0.758	**0.616**
Wavelet-Demy Train	R	0.934	0.976	0.977	0.983
	RMSE	237.157	144.794	143.518	123.956
	MAE	176.953	110.228	107.859	95.326
	RAE	0.327	0.203	0.199	0.176
	MAPE	9.814	6.030	5.973	5.437
	E	0.873	0.953	0.954	0.965
	I_A_	0.965	0.988	0.988	0.991
	PI	1.000	0.760	0.754	0.712
Wavelet-Bior Train	R	0.926	0.956	0.975	0.982
	RMSE	252.313	196.290	148.253	126.166
	MAE	186.700	151.103	115.537	97.569
	RAE	0.344	0.279	0.213	0.180
	MAPE	10.175	8.270	6.406	5.568
	E	0.857	0.913	0.950	0.964
	I_A_	0.960	0.977	0.987	0.991
	PI	1.000	0.872	0.754	0.699

Significant values are in bold.

With regard to Table 11, the W-GRNN outcomes reveal the best combination is Combo 1 for both mother wavelets (i.e., Dmey and Bior6.8). Statistically, the best combination of W-GRNN-Dmey and W-GRNN-Bior6.8 are (R = 0.811, RMSE = 214.160, MAPE = 9.055, E = 0.634, and PI = 0.937) and Comb 1 (R = 0.810, RMSE = 219.231, MAPE = 9.238, E = 0.617, and PI = 0.960, correspondingly. As a result, the best model is considered W-GRNN-Dmey with the PI equal to 0.937.

**Table 11:** Statistic metrics obtained by W-GRNN model to forecast the EC parameter for all combinations.

Model	Criteria	Combination			
		Combo 1	Combo 2	Combo 3	Combo 4
Wavelet-Demy Test	R	**0.811**	0.785	0.797	0.790
	RMSE	**214.160**	224.622	220.293	225.909
	MAE	**166.059**	181.860	179.529	185.436
	RAE	**0.598**	0.655	0.647	0.668
	MAPE	**9.055**	9.727	9.442	9.677
	E	**0.634**	0.597	0.612	0.592
	I_A_	**0.879**	0.869	0.881	0.881
	PI	**0.937**	0.993	0.974	0.996
Wavelet-Bior Test	R	**0.810**	0.791	0.792	0.789
	RMSE	**219.122**	221.946	222.328	225.607
	MAE	**171.231**	180.733	180.477	184.291
	RAE	**0.617**	0.651	0.650	0.664
	MAPE	**9.238**	9.560	9.598	9.676
	E	**0.617**	0.607	0.605	0.594
	I_A_	**0.876**	0.877	0.875	0.878
	PI	**0.960**	0.987	0.988	1.000
Wavelet-Demy Train	R	0.933	0.959	0.974	0.986
	RMSE	251.635	200.526	159.135	115.105
	MAE	185.355	148.077	114.561	77.823
	RAE	0.342	0.273	0.211	0.144
	MAPE	10.074	7.874	5.902	3.837
	E	0.857	0.909	0.943	0.970
	I_A_	0.956	0.973	0.984	0.992
	PI	1.000	0.868	0.756	0.639
Wavelet-Bior Train	R	0.957	0.969	0.966	0.982
	RMSE	204.698	176.366	182.512	134.219
	MAE	146.654	128.769	133.566	93.273
	RAE	0.271	0.238	0.246	0.172
	MAPE	7.787	6.737	7.003	4.669
	E	0.906	0.930	0.925	0.959
	I_A_	0.972	0.980	0.978	0.989
	PI	1.000	0.920	0.939	0.775

Significant values are in bold.

**Figure 12:**
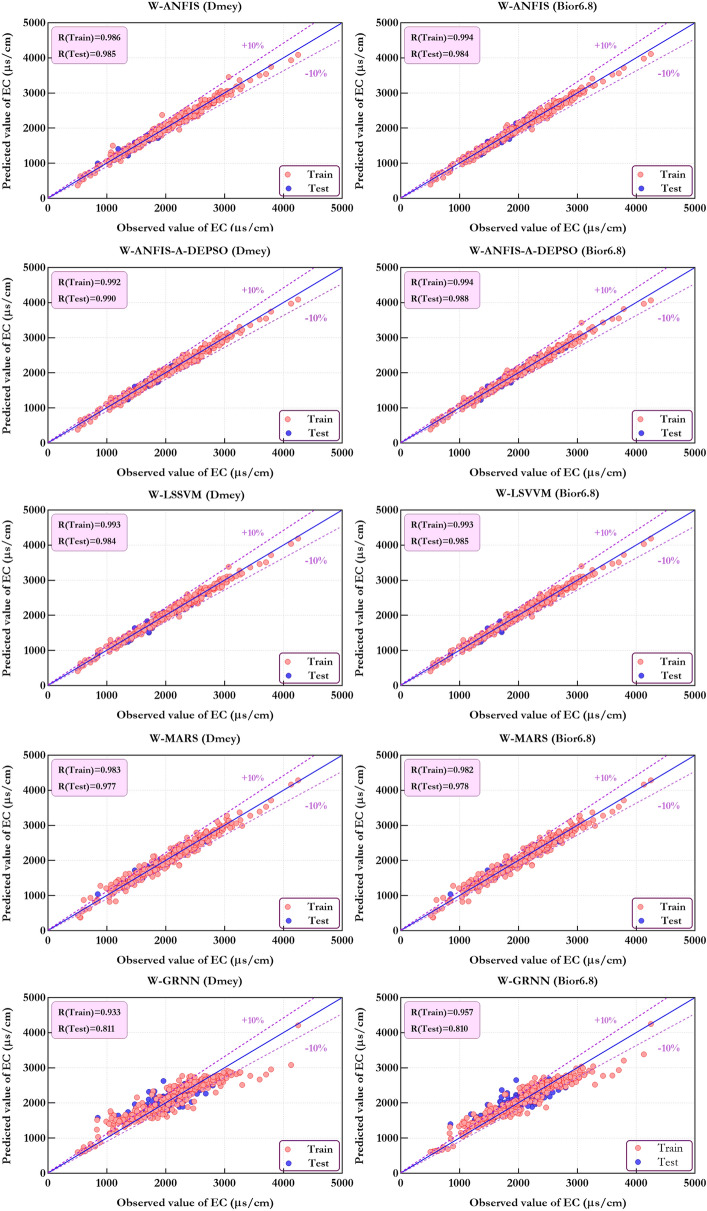
Compare estimated and measured values of EC utilizing the W-ML models in the form of a scatter plot.

Figure 12 depicts the comparison's results of predicted $EC_{t}$ and observed $EC_{t}$ being carried out by five W-ML models in the best combination of each mother wavelet. As can be observed, W-ANFIS-A-DEPSO-Dmey (Combo 4) outperforms compared to the W-ANFIS-A-DEPSO with Bior6.8 mother wavelet and the four others with all mother wavelets. To add to it, the proportion of errors concerning W-ANFIS-A-DEPSO-Dmey (Combo 4) accounted for under 10% for the majority of predicted values. Comparing this figure with Fig. 10, it is clearly observed that the hybrid model W-ANFIS-A-DEPSO can improve the correlation coefficient (R = 0.988) up to 52% compared to the standalone ANFIS-A-DEPSO (R = 0.672) during the test period.

**Figure 13:**
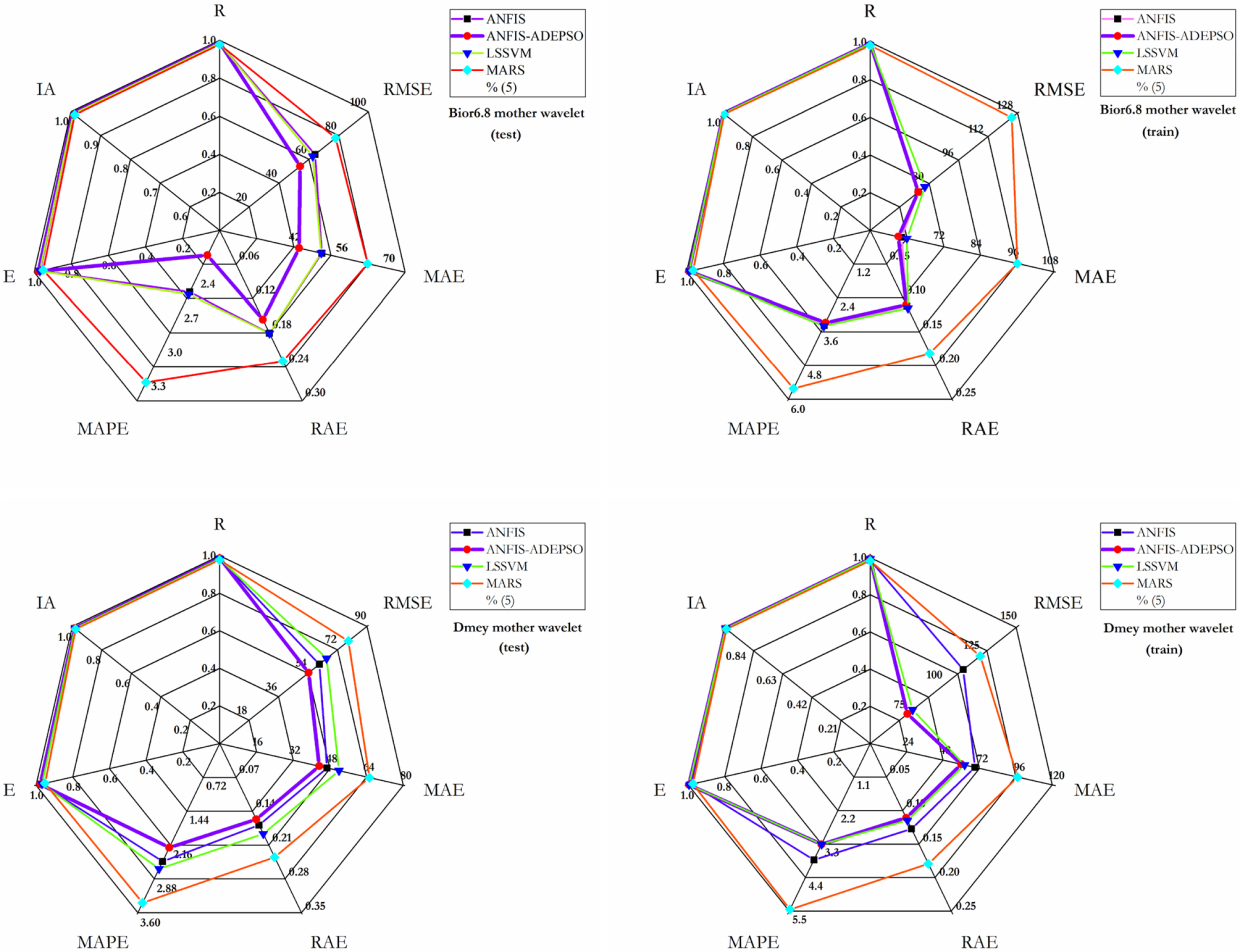
Spider plots of seven performance criteria for prediction of EC using all ML models for Bior6.8 (upper panel) and Dmey (lower panel) in training and testing stages.

The spider plot based on seven factors for the top four models along with the best combination of input variables is displayed in Fig. 13. In fact, according to the mentioned diagram, the more the values of "R, I_A_ and E_L,M_" and "RMSE, MAE, RAE, and MAPE" obtained by each model become closer to the value 1 and to the center of the diagram, respectively, the more the model is reliable. According to Fig. 13 (lower panel), the most effective model to rise the accuracy of forecasting the $EC_{t}$ is W-ANFIS-A-DEPSO-Demy with the largest R, I_A_, and E_L,M_, and smallest RMSE, RAE, and MAPE for both training and testing stages. On the other hand, Fig. 13 (upper panel) depicts the W-ANFIS-A-DEPSO-Bior6.8-C4 with the highest R, I_A_, and E_L,M_, and lowest RMSE, RAE, and MAPE have the most significant impact on the accuracy of $EC_{t}$ prediction for both training and testing stages.

**Figure 14:**
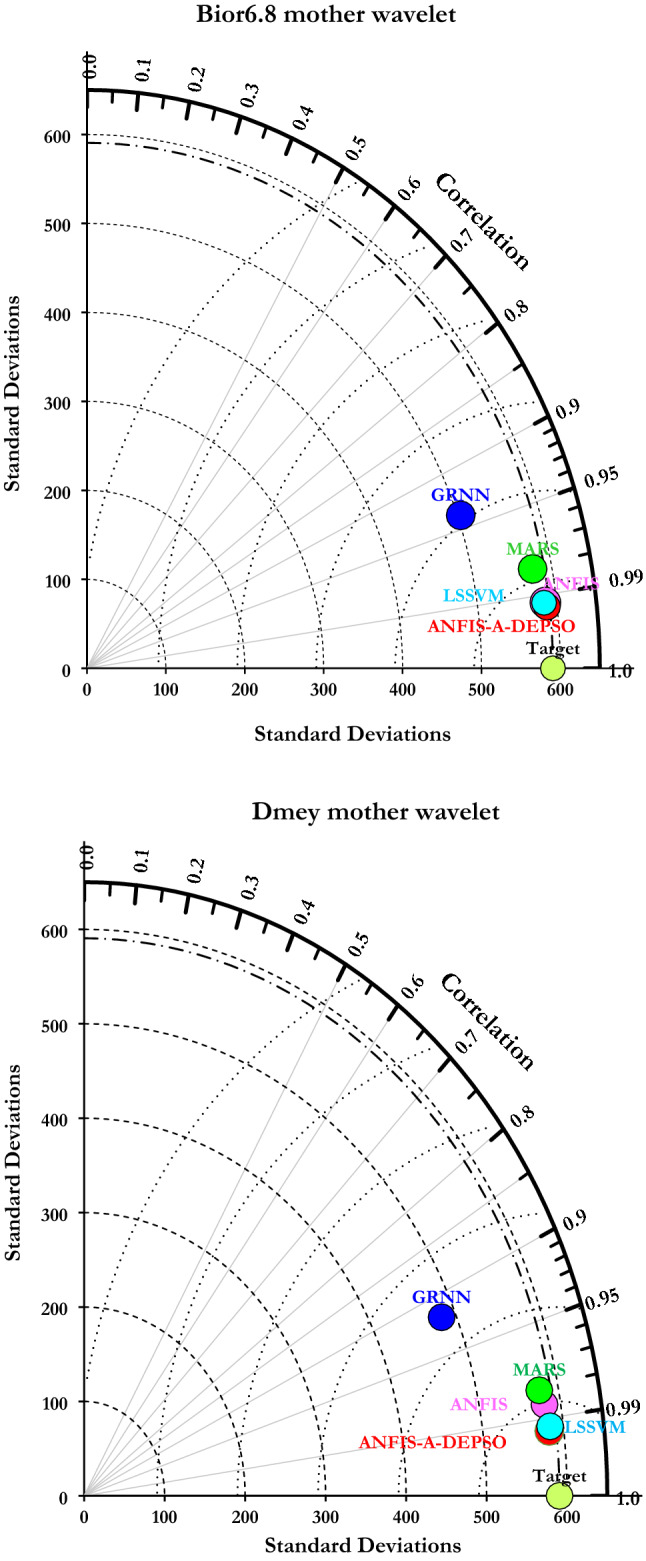
Taylor diagram of all ML models for Bior6.8 (upper panel) and Dmey (lower panel).

The correlation coefficient (R) was figured based on the Taylor diagram to assess the overall ability of the models, with it providing the models' efficiency in detail^5,11^. According to the *R* and standard deviation, the diagram demonstrated a more perceptible and persuasive connection between predicted and observed WQ parameters. The Taylor diagram illustrated in Fig. 14 associated the current monthly EC with Bior6.8 mother wavelet (Upper panel) and EC with Dmey mother wavelet (Lower panel) for all ML models. As a result, W-ANFIS-A-DEPSO has the most suitable performance for EC prediction compared with the other models and is the closest model to the target point.

### Compare the performance of all ML models

The contrastive analysis is provided in this section to determine the best model. Consequently, five ML models along with two mother wavelets and four combinations of input variables are under review to forecast the EC in this research. As mentioned before, the W-ANFIS-A-DEPSO-C4 (Dmey), W-ANFIS-C4 (Dmey), W-LSSVM-C4 (Bior6.8), W-MARS-C1 (Dmey), and W-GRNN-C1 (Dmey) have the better performance amongst all models for $EC_{t}$ prediction.

**Figure 15:**
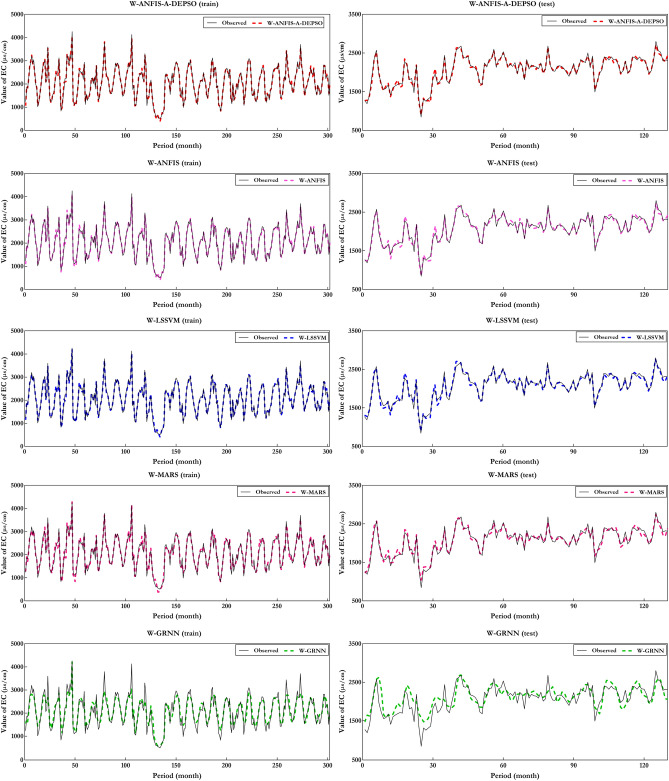
Compare the physical trend of the best performance of all ML models.

Figure 15 demonstrates the physical trend of five methods to further address their abilities, which results in the disability of standalone ML models in prediction for the $EC_{t}$. Since there are high variations and the characteristic non-linear correlation between the WQ parameters, making a steady model through ANFIS-A-DEPSO, ANFIS, LSSVM, MARS, and GRNN is sophisticated matter. In turn, the aim is to enhance five meticulous ML models concerning wavelet theorem and assess the impact of wavelet transform joined with ML models for EC prediction. According to Fig. 15 W-MLs have the edge over standalone ML models without wavelets in terms of efficiency.

**Figure 16:**
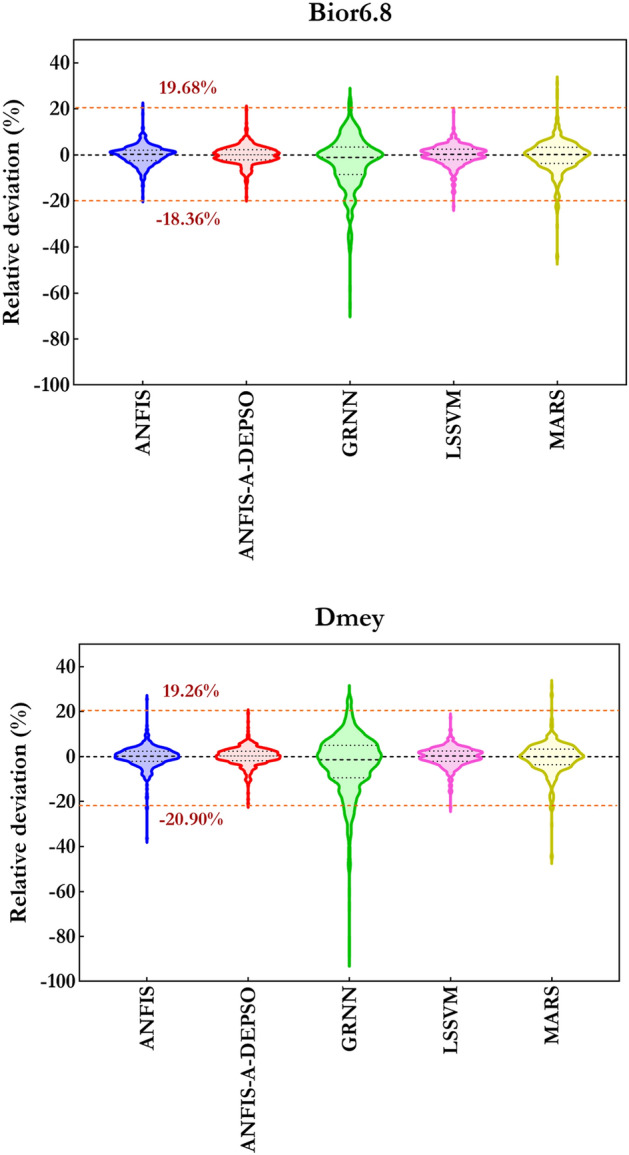
Relative deviation to forecast the EC using ML models coupled with Bior6.8 (upper) and Dmey (lower) mother wavelets.

Eventually, Fig. 16, in which the relative deviation (RD) to predict the $EC_{t}$ by W-ANFIS-A-DEPSO, W-ANFIS, W-LSSVM, W-MARS, and W-GRNN in diverse combinations is illustrated, confirms that the W-ANFIS-A-DEPSO-C4 with the RD in the domain of [− 43.89, 29.60] has the superior ability to predict the $EC_{t}$ amongst other models.

## Comparison of W-ANFIS-A-DEPSO with hybrid models

To further investigation of the proposed W-ANFIS-A-DEPSO efficiency, this section compares the performance of proposed model with three hybrid models, comprising W-ANFIS-PSO [i.e., hybrid of W-ANFIS with particle swarm optimization^92^ (W-ANFIS-PSO)], W-ANFIS-GWO [i.e., hybrid of W-ANFIS with grey wolf optimizer^93^ (W-ANFIS-GWO)], and W-ANFIS-WOA [i.e., hybrid of W-ANFIS with whale optimization algorithm^94^ (W-ANFIS-WOA)]. To implement a fair comparison, the population size and the maximum number of iterations (MaxIt) are equal to 50 and 300 for all hybrid models respectively, except for ANFIS-A-DEPSO, which is equal to 50. In fact, by choosing the value of 50 for MaxIt, we try to show that the proposed model can provide better performance than other models in a much smaller number of iterations. Table 12 reports the parameter settings of all methods. According to the selected hybrid models, the PSO, GWO, and WOA do not have any parameter settings. For instance, PSO uses a weighted factor ($w$), decreasing with a linear relationship, to damp its velocity, so it does not need to be set. It should be noted that the values of parameters c1 and c2 are constant. In Refs.^74,92^ their values are recommended equally to 1.5 for both of them. This is true of the other two methods as well (i.e., W-ANFIS-GWO and W-ANFIS-WOA). In this section, all hybrid models were applied to predict the EC parameter using the Combo 4-Dmey as the input, because this combination has a better performance for the ANFIS model based on previous sections.

**Table 12:** Control parameters of the LSSVM, MARS, and GRNN models.

Model	Parameters
W-ANFIS-A-DEPSO	$\beta =50$ and $\delta =1$
W-ANFIS-GWO	$a=2-iter.(2)/MaxIt$
W-ANFIS-WOA	$a1=2-iter.(2)/MaxIt$ $a2=-1-iter.(-1)/MaxIt$
W-ANFIS-PSO	$c1=c2=1.5$ and $w=0.9-0.5.(\frac{iter}{MaxIt})$

**Figure 17:**
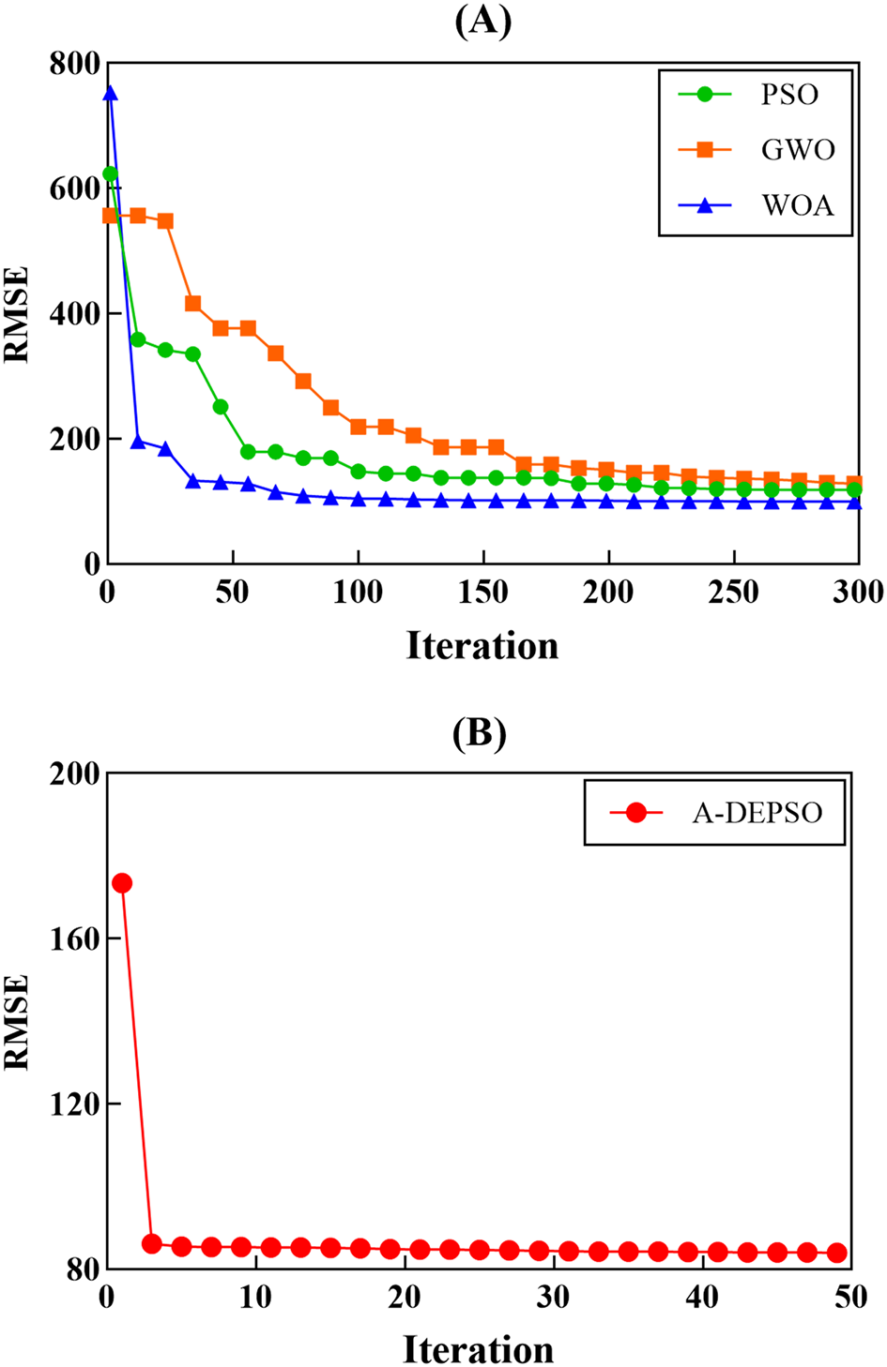
Convergence graphs of (**A**) PSO, GWO, WOA, and (**B**) A-DEPSO.

Figure 17 displays the convergence graphs of all hybrid models to predict the EC parameter. From the figure, it can be clearly seen that the proposed model can converge to a lower value (83.955) compared with the other hybrid models. Also, the proposed model can achieve a better value of RMSE at less than 10 iterations, while the other hybrid models cannot even converge to a suitable solution after 50 iterations. This confirms the proposed model's superiority compared to the other hybrid models again.

Tables 13 and 14 give the statistical outcomes of the WANFIS-A-DEPSO and three other hybrid models to predict the EC parameter for both training and testing stages. According to these tables, the proposed W-ANFIS-A-DEPSO can provide better results in terms of RMSE (train: 83.955, test: 51.193), MAE (train: 65.822, test: 41.870), RAE (train: 0.121. test: 0.1509), and MAPE (train: 3.607, test: 2.1427) compared with the other hybrid models. Simply put, due to the fact that the proposed model using powerful exploration and exploitation mechanisms and adaptive parameters to better transit from global to local search, it can present more accurate outcomes fast compared to the other hybrid models.

**Table 13:** Compare W-ANFIS-A-DEPSO with three hybrid models in training stage.

	W-ANFIS-A-DEPSO	W-ANFIS-PSO	W-ANFIS-GWO	W-ANFIS-WOA
RMSE	83.955	118.040	128.617	99.803
MAE	65.822	75.005	80.038	74.224
RAE	0.121	0.138	0.148	0.137
MAPE	3.607	4.224	4.402	4.115

**Table 14:** Compare W-ANFIS-A-DEPSO with three hybrid models in testing stage.

	W-ANFIS-A-DEPSO	W-ANFIS-PSO	W-ANFIS-GWO	W-ANFIS-WOA
RMSE	51.1934	54.8850	57.9785	55.9158
MAE	41.8701	43.7943	47.0106	44.8192
RAE	0.1509	0.1578	0.1694	0.1615
MAPE	2.1427	2.2905	2.4507	2.3450

**Figure 18:**
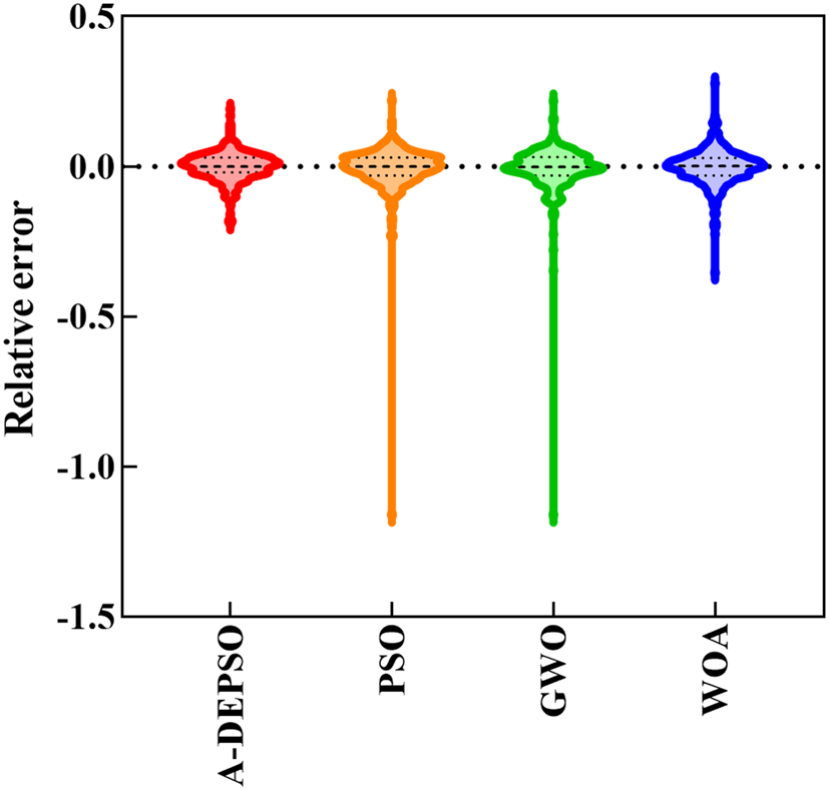
Relative errors calculated by all hybrid models.

Figure 18 depicts all hybrid models' relative errors (REs). Regarding the figure, the proposed model can estimate the EC with a less RE range ([− 0.190, 0.191]) compared with PSO ([− 1.160, 0.22]), GWO ([− 1.610, 0.22]), and WOA ([− 0.353, 0.276]). The proposed model can predict the EC with high accuracy based on these results compared with the other hybrid methods.

## Conclusion

By designing a promising model called wavelet-ANFIS-A-DEPSO with two mother wavelets (Dmey and bior6.8), *EC*_*t*_ prediction can be made on a monthly basis in surface water. In fact, a powerful optimization method, A-DEPSO, was developed to increase the ability of ANFIS models. The A-DEPSO is a hybrid of DE and PSO with a boost of exploration and exploitation and two adaptive parameters. In addition, a novel crossover with adaptive parameters was used to increase the diversity of the population. Moreover, a refreshing operator was implemented to raise the chance of escaping from local solutions. Four ML models (i.e., ANFIS, LSSVM, MARS, and GRNN) were operated to forecast the EC with the purpose of evaluating the proposed model's efficiency. Besides, standalone ML models were utilized to assess the predictive ability of all W-ML models for the EC water quality parameter via some metrics and validation manner. Consequently, the monthly time series of Q and EC were operated during 36 years in the Maroon river within two and four time-lagged correspondingly. Indeed, these two and four time-lagged were detected by statistical procedures, and three decomposing levels were used for each mother wavelets.

More specifically, the best subset regression analysis was considered to detect the best input combination of EC prediction. The gained outcomes of the ML model without wavelet in all combinations proved that the ANFIS-A-DEPSO model in Combo 4 had the striking ability in the prediction of EC (PI = 0.832) on a monthly basis. To add to it, in Combo 4 (PI = 0.887), Combo 1 (PI = 0.964), Combo 4 (PI = 0.890), and Combo 1 (PI = 0.948), the ANFIS, LSSVM, MARS, and GRNN models correspondingly showed the superior performance for EC prediction. In addition, seven metrics obtained by the ANFIS-A-DEPSO as the best model are R = 0.672, RMSE = 275.404, MAE = 198.092, RAE = 0.714, MAPE = 10.956, E = 0.394, and I_A_ = 0.801.

More importantly, W-ML models improved the certainty of EC modelling. The Dmey, jointed with ANFIS-A-DEPSO, ANFIS, MARS, and GRNN models to predict EC, proved the noticeable and best advancement in terms of the accuracy level of simulation, albeit Bior 6.8 showed appropriate performance. On the other hand, when Bior6.8 joined with LSSVM brought about a more suitable performance compared to Dmey.

Regarding monthly *EC*_*t*_ prediction, it is clear that W-ANFIS-A-DEPSO-Dmey in Combo 4 had the best efficiency (PI = 0.485), while W-ANFIS-Dmey model in Combo 4 (PI = 0.517) also proved the reliable ability, followed by W-LSSVM-Bior6.8 in Combo 4 (PI = 0.572), W-MARS-Dmey in Combo 4 (PI = 0.612), and W-GRNN-Dmey in Combo 4 (PI = 0.937) correspondingly. Furthermore, by comparing the seven statistical metrics obtained by W-ANFIS-A-DEPSO-Dmey (R = 0.988, RMSE = 53.841, MAE = 42.941, RAE = 0.155, MAPE = 2.192, E = 0.977, and I_A_ = 0.994) and W-ANFIS-Dmey (R = 0.985, RMSE = 60.295, MAE = 46.328, RAE = 0.167, MAPE = 2.484, E = 0.971, and I_A_ = 0.993), it can be clearly determined that the proposed model has a better efficiency than the W-ANFIS model. Moreover, according to the graphical analysis (i.e., scatter plots, time series plots, Taylor diagram, and violon graph), it is evident that the proposed model can predict the EC parameter more accurate and reliable than the other models.

Furthermore, the suggested model was compared to three hybrid models (W-ANFIS-PSO, W-ANFIS-GWO, and W-ANFIS-WOA) to evaluate its effectiveness. The findings show that the suggested model is more accurate in terms of RMSE (train: 83.955, test: 51.193) and MAPE (train: 3.607, test: 2.1427) than the other models.

To sum up, from what had been addressed in all ML-based models, it is obvious that the W-ANFIS-A-DEPSO, a supplementary model, is able to predict the EC accurately. As a suggestion, firstly, it would be operated as an ensemble multi-wavelet model in order to use wavelets simultaneously. Secondly, designing an ensemble ANFIS-based method could have a positive impact on WQPs prediction in surface water, which may lead to accumulating the merits of each supplementary procedure. Finally, it can be applied to other optimization methods to optimize the main parameters of ANFIS model^95–97^.

## Data Availability

The data that support the findings of this study are available from the corresponding author upon reasonable request.
